# Small molecule induces mitochondrial fusion for neuroprotection via targeting CK2 without affecting its conventional kinase activity

**DOI:** 10.1038/s41392-020-00447-6

**Published:** 2021-02-19

**Authors:** Ke-Wu Zeng, Jing-Kang Wang, Li-Chao Wang, Qiang Guo, Ting-Ting Liu, Fu-Jiang Wang, Na Feng, Xiao-Wen Zhang, Li-Xi Liao, Mei-Mei Zhao, Dan Liu, Yong Jiang, Pengfei Tu

**Affiliations:** 1 State Key Laboratory of Natural and Biomimetic Drugs, School of Pharmaceutical Sciences, Peking University, Beijing 100191, China; 2 Proteomics Laboratory, Medical and Healthy Analytical Center, Peking University Health Science Center, Beijing 100191, China

**Keywords:** Target identification, Target identification

## Abstract

Mitochondrial fusion/fission dynamics plays a fundamental role in neuroprotection; however, there is still a severe lack of therapeutic targets for this biological process. Here, we found that the naturally derived small molecule echinacoside (ECH) significantly promotes mitochondrial fusion progression. ECH selectively binds to the previously uncharacterized casein kinase 2 (CK2) α′ subunit (CK2α′) as a direct cellular target, and genetic knockdown of CK2α′ abolishes ECH-mediated mitochondrial fusion. Mechanistically, ECH allosterically regulates CK2α′ conformation to recruit basic transcription factor 3 (BTF3) to form a binary protein complex. Then, the CK2α′/BTF3 complex facilitates β-catenin nuclear translocation to activate TCF/LEF transcription factors and stimulate transcription of the mitochondrial fusion gene Mfn2. Strikingly, in a mouse middle cerebral artery occlusion (MCAO) model, ECH administration was found to significantly improve cerebral injuries and behavioral deficits by enhancing Mfn2 expression in wild-type but not CK2α′^+/−^ mice. Taken together, our findings reveal, for the first time, that CK2 is essential for promoting mitochondrial fusion in a Wnt/β-catenin-dependent manner and suggest that pharmacologically targeting CK2 is a promising therapeutic strategy for ischemic stroke.

## Introduction

Mitochondria, which are subcellular organelles responsible for energy production, are considered to play a crucial role in ischemic stroke.^1–3^ Mitochondrial impairment leads to excessive oxidative stress, mitochondrial membrane potential (MMP) decreases, and mitochondrial DNA (mtDNA) release; therefore, mitochondrial quality control is crucial for maintaining a healthy mitochondrial population and thus preserving proper cell function.^4,5^ Nonetheless, the current treatment options for modulating mitochondrial homeostasis during cerebral ischemia remain limited.

Mitochondria are highly dynamic organelles that continuously undergo fusion and fission.^6,7^ Mitochondrial fusion/fission balance is necessary for cell adaptation to changing environments.^8^ Mechanistically, fusion contributes to restoring mitochondrial function by enabling mixing of the contents of partially damaged mitochondria as a form of complementation.^9^ In addition, fission may help to isolate and clear damaged mitochondrial segments via macroautophagy in order to generate new, healthy mitochondria.^10,11^ Thus far, several functional proteins that control mitochondrial fission/fusion dynamics have been identified, such as fission protein 1 (Fis1), mitochondrial fission factor (Mff), dynamin-related protein 1 (Drp1), and mitofusin 1/2 (Mfn1/2).^12–14^ However, there is still a severe lack of effective pharmacological strategies, particularly strategies involving novel therapeutic targets, that can be used to modulate mitochondrial fission/fusion progression.^15^

Casein kinase 2 (CK2), a highly evolutionarily conserved serine/threonine kinase, is a stable tetrameric complex consisting of two catalytic (α and α′) subunits and two regulatory (β) subunits.^16,17^ CK2 is involved in a variety of crucial cell events, including gene transcription, signal transduction, cytoskeletal structure regulation, and cell adhesion.^18–20^ Notably, previous reports have suggested that CK2 widely contributes to neuronal survival and differentiation by mediating a variety of signaling proteins, such as NF-κB,^21^ SCF^(cyclin F)^ E3 ligase,^22^ PACSIN,^23^ and N-methyl-d-aspartate receptor (NMDAR),^24^ indicating that CK2 may be a versatile regulator of central nervous system injury and repair. Given the essential biological roles of CK2 in signaling pathways related to neurological function, whether CK2 is a potential target for mitochondrial fusion/fission dynamics control in neuronal cells deserves considerable attention.

Notably, in this study, we found that the natural small molecule echinacoside (ECH) noticeably promotes the mitochondrial fusion process by directly targeting the CK2 α′ subunit (CK2α′). Mechanistic studies showed that ECH selectively binds to CK2α′ to recruit basic transcription factor 3 (BTF3) as a substrate to form a binary protein complex. Then, the CK2/BTF3 complex promotes Wnt/β-catenin signal activation and subsequent transcription of the mitochondrial fusion-related gene *Mfn2*, resulting in remarkable neuroprotection against cerebral ischemia.

In summary, for the first time, we have built a conceptual framework to explain the fundamental role of CK2 in promoting the mitochondrial fusion process in a Wnt/β-catenin-dependent manner and have shed light on CK2 as a previously undiscovered therapeutic target for ischemic stroke.

## Results

### ECH promotes mitochondrial fusion for neuroprotection

**Fig. 1:**
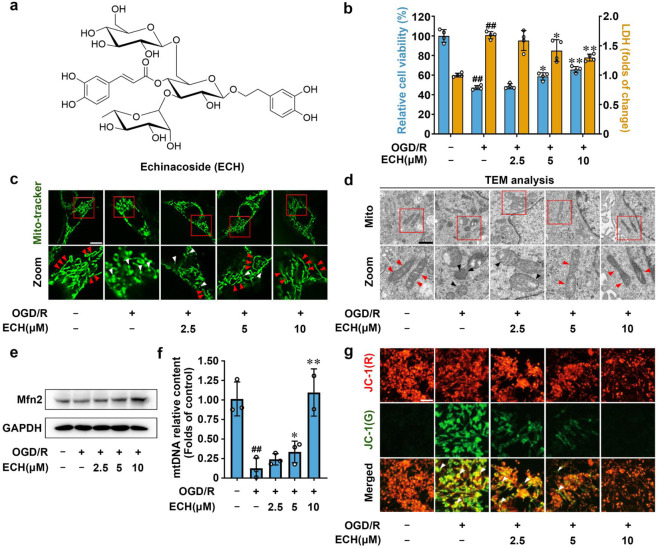
Small-molecule ECH exerts neuroprotection via promoting mitochondrial fusion. **a** Chemical structure of echinacoside (ECH). **b** ECH increased cell viability and decreased LDH release against OGD/R-induced injury in PC12 cells. **c** ECH promoted mitochondrial fusion against OGD/R insult in PC12 cells. Arrows (red) indicate branched healthy mitochondria. Arrows (white) indicate spherical dysfunctional mitochondria (scale bar: 20 μm). **d** ECH reversed the fragmented mitochondria by promoting mitochondrial fusion, which was analyzed by transmission electron microscopy (TEM). Arrows (red) indicate healthy mitochondria. Arrows (black) indicate damaged mitochondria (scale bar: 0.5 μm). **e** ECH (10 μM) increased Mfn2 protein expression, which was analyzed by western blot. **f** ECH increased mitochondrial DNA (mtDNA) content against OGD/R-induced injury. **g** ECH blocked OGD/R-induced decrease in mitochondrial membrane potential (MMP), which was detected by JC-1 staining. Arrows indicate depolarized mitochondria (scale bar: 100 μm). Data are expressed as the mean ± SD. ^##^*P* < 0.01 vs. control group, *P < 0.05, **P < 0.01 vs. OGD/R group

We screened a natural product library containing 1150 small molecules for their activity against oxygen-glucose deprivation/reperfusion (OGD/R) insult and ultimately found that ECH exerted a significant neuroprotective effect on PC12 cells by increasing cell viability and inhibiting lactate dehydrogenase (LDH) release (Fig. 1a, b). Cell organelles are important for maintaining cell survival, and each organelle exhibits a specific function in modulating cell status. We thus investigated whether ECH exerted a protective effect on PC12 cells by regulating the functions of specific cell organelles. To this end, we examined the morphological properties of mitochondria, the endoplasmic reticulum (ER), the Golgi apparatus, and lysosomes using different organelle-specific fluorescence dyes. Notably, we observed that OGD/R insult induced significant mitochondrial fission and fragmentation; however, this process was effectively attenuated by ECH treatment. Other cell organelles were not significantly affected by ECH treatment (Fig. 1c, Supplementary Fig. 1a–e). This finding was further confirmed by transmission electron microscopy (TEM) analysis, which showed that ECH had a pronounced promoting effect on mitochondrial integrity (Fig. 1d).

Previous studies have shown that the *Mfn2* gene encodes a mitochondrial membrane protein that plays a central role in regulating mitochondrial fusion.^25^ As shown in Fig. 1e, ECH significantly increased Mfn2 protein expression with or without OGD/R insult but did not obviously affect mitochondrial Fis1 (Supplementary Fig. 1f, g). Moreover, ECH increased *Mfn2* gene transcription, increasing its mRNA levels (Supplementary Fig. 1h). Thus, we speculate that ECH may promote mitochondrial fusion by facilitating Mfn2 expression. Since mitochondrial fusion plays a critical role in maintaining mitochondrial integrity and function, we next performed mtDNA and MMP analyses. Our data showed that OGD/R significantly decreased mtDNA content and induced MMP collapse and that both of these changes were notably attenuated by ECH treatment (Fig. 1f, g). Taken together, these observations indicate that ECH markedly promotes the mitochondrial fusion process to maintain normal mitochondrial biological function.

### CK2α′ is a direct cellular target of ECH

**Fig. 2:**
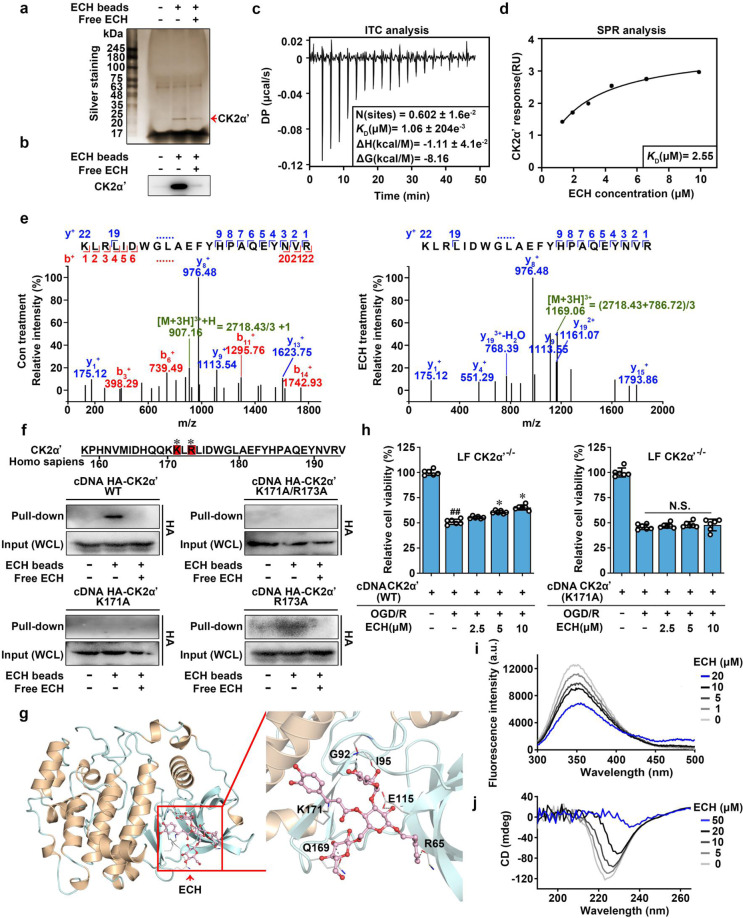
Casein kinase 2α′ is a direct cellular target of ECH. **a** Identification of cellular target of ECH using pull-down technology coupled with LC–MS/MS. PC12 lysate was incubated with control beads or ECH beads. The binding proteins were detected by SDS–PAGE, followed by silver staining and LC–MS/MS analysis. **b** The binding proteins were detected by western blot. **c** Calorimetric titration of ECH with CK2α′. **d** ECH interacted with CK2α′, which was detected by SPR analysis. **e** LC–MS/MS analysis of covalently modified peptide by ECH (10 μM) in CK2α′ protein. **f** CK2α′ (K171A), but not CK2α′ (R173A) is responsible for ECH binding to CK2α′. HEK293T cells were transfected with HA-tagged CK2α′ and its mutant plasmids. The Lys and Arg labeled by * were potential modification sites. **g** Docking analysis of ECH covalent binding mode to CK2α′. **h** ECH increased cell viability against OGD/R-induced injury in CK2α′^−/−^ lung fibroblasts (LF), which were supplemented with CK2α′ but not CK2α′ mutant plasmids. **i** Fluorescence spectroscopy analysis of the conformational change of CK2α′ with ECH. **j** CD spectra analysis for ECH-mediated CK2α′ conformational change. Data are expressed as the mean ± SD. ^##^*P* < 0.01 vs. control group, *P < 0.05, **P < 0.01 vs. OGD/R group

Cellular target is the molecular basis of ECH-mediated regulation of mitochondrial fusion. We thus sought to identify the direct cellular target protein of ECH using small-molecule affinity-based chromatography analysis.^26^ We prepared ECH-conjugated epoxy-activated Sepharose beads (ECH beads) to capture binding proteins of ECH and then performed silver staining and liquid chromatography (LC)–tandem mass spectrometry (MS/MS) analysis. As shown in Fig. 2a, b, an obvious protein band was identified as CK2α′. To confirm the direct interaction of ECH with CK2α′, we performed isothermal titration calorimetry (ITC) analysis to characterize the thermodynamics of ECH with CK2α′ binding. The results showed that ECH bound to CK2α′ in a 1:1 ratio with a dissociation constant (*K*_D_) of 1.06 μM (Fig. 2c, Supplementary Fig. 2a), demonstrating a direct interaction between ECH and CK2α′. This observation was supported by the results of a surface plasmon resonance (SPR) assay (*K*_D_ = 2.55 μM), indicating that ECH strongly interacts with CK2α′ (Fig. 2d).

Next, we explored the binding selectivity of ECH with different CK2 subunits, including CK2α, CK2α′, and CK2β, using a nanoscale differential scanning fluorimetry (NanoDSF) assay. As shown in Supplementary Fig. 2b, ECH concentration-dependently increased the thermal stability of CK2α′ by increasing Δ*T*_m_ by 1.2 °C; however, ECH did not significantly affect the Δ*T*_m_s of CK2α and CK2β, suggesting that ECH selectively binds to CK2α′. Quantitative analysis of the interactions of ECH with different CK2 subunits was performed using cellular thermal shift assay (CETSA) technology. As shown in Supplementary Fig. 2c, the lysates treated with ECH showed a higher abundance of CK2α′ than CK2α or CK2β from 50 to 60 °C, suggesting that ECH directly engages with CK2α′ to exert ligand-dependent stabilization. This result was also confirmed by drug-affinity responsive target stability (DARTS) assay. We also found that ECH concentration-dependently reduced proteolysis of CK2α′ but not CK2α or CK2β (Supplementary Fig. 2d). Finally, these observations were supported by the SPR assay, which revealed that the *K*_D_ values of CK2α (45.80 μM) and CK2β (48.60 μM) with ECH were more than 20 times larger than that of CK2α′ with ECH (Fig. 2d, Supplementary Fig. 2e). Collectively, these data suggest that CK2α′ is a direct cellular target of ECH and that ECH selectively binds to CK2α′ rather than CK2α or CK2β.

### ECH covalently modifies the K171 site of CK2α′

To explore the mechanism of the interaction of ECH with CK2α′, we investigated the ECH-CK2α′ complex using LC–MS/MS.^27^ As shown in Fig. 2e, ECH was found to covalently modify the peptide (residues 158–192) on lysine (K) 171 or arginine (R) 173 site. Since ECH contains an α,β-unsaturated carbonyl group, we hypothesized that ECH may react covalently with the amino group of K or R on CK2α′ (Supplementary Fig. 3). To test this hypothesis, we mutated K171 into alanine (A) 171, R173 into A173, and K171/R173 into A171/A173 (double mutation). Pulldown assays showed that both K171 and K171/R173 mutations significantly attenuated the binding capacity of ECH for CK2α′, but R173 mutation exhibited a weaker effect (Fig. 2f), indicating that K171 is a potential binding site of ECH on CK2α′. Furthermore, ITC analysis revealed that the *K*_D_ values of ECH to CK2α′ (R173A) was 413 nM. However, no binding of ECH to CK2α′ (K171A) (Supplementary Fig. 3b, c). Taken together, these results demonstrate that ECH directly binds to CK2α′ through 171 lysine. Covalent docking analysis against the predicted active pocket containing K171 on CK2α′ further confirmed that ECH binds to CK2α′ by forming a covalent bond (1.5 Å) with K171 (Fig. 2g, Supplementary Fig. 3d, e). Additionally, to observe the conformational change of CK2α′ upon ECH binding, we superposed our docking model to previously published structure (PDB ID: 5OOI). Around ECH-binding pocket, we found side chain of Lys171 contrarotated to form a covalent bond with ECH in docking model. Thus, we speculated that ECH induced a conformational change of Lys171 to assist their covalent attachment (Supplementary Fig. 3f). Moreover, hydrogen bond interactions were also observed between ECH and multiple CK2α′ residues, including leucine (R) 65, isoleucine (I) 95, glutamic acid (E) 115, glutamine (Q) 169, and glycine (G) 92. These hydrogen bond interactions may provide fundamental conditions for the covalent reaction of ECH with CK2α′. Next, to validate the essential biological role of K171, we transfected CK2α′ and CK2α′(K171A) plasmids into primary cultured CK2α′^−/−^ mouse fibroblasts and observed that the ECH-dependent cytoprotective effect was markedly reversed by reconstitution with CK2α′ but not with the mutant form CK2α′ (K171A) (Fig. 2h), indicating that K171 on CK2α′ is crucial for ECH binding and resulting neuroprotection.

Furthermore, to explore whether ECH influences CK2 function, we performed tryptophan fluorescence scanning of CK2α′. As shown in Fig. 2i, CK2α′ fluorescence intensity was obviously decreased by ECH, suggesting that ECH dynamically induces conformational changes in CK2α′. Moreover, CK2α′ secondary structure was evaluated by CD spectroscopy analysis, and our results showed that ECH-induced pronounced molar ellipticity value decreases at 208 and 222 nm, suggesting a concentration-dependent reduction in protein helicity. Furthermore, we evaluated the regulatory effect of ECH on the catalytic activity of CK2. Interestingly, ECH did not affect CK2-dependent phosphorylation on the classic substrate peptide (Supplementary Fig. 4), indicating that ECH does not directly regulate the catalytic activity of CK2, at least not through the classic phosphorylation motif. Overall, these results suggest that K171 site on CK2α′ is directly bound and covalently modified by ECH, which induces conformational regulation of CK2α′.

### CK2α′ plays a fundamental role in modulating mitochondrial fusion

**Fig. 3:**
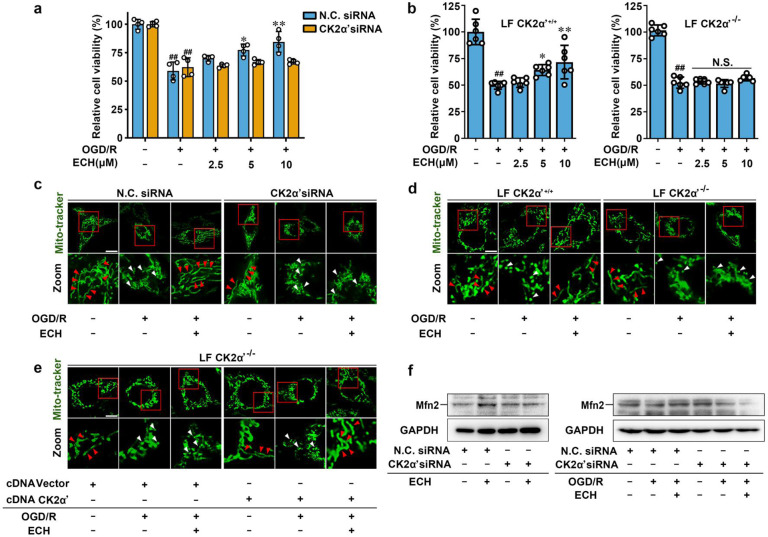
CK2α′ plays a fundamental role in modulating mitochondrial fusion. **a** CK2α′ knock-down reversed ECH-dependent increase in cell viability against OGD/R insult, which was detected by MTT assay. **b** ECH increased cell viability against OGD/R-induced injury in CK2α′^+/+^ lung fibroblasts, which was significantly suppressed in CK2α′^−/−^ cells. **c** ECH-induced mitochondrial fusion was suppressed in CK2α′ knock-down cells, which was indicated by Mito-tracker staining. Arrows (red) indicate branched healthy mitochondria. Arrows (white) indicate spherical dysfunctional mitochondria (scale bar: 20 μm). **d** ECH-induced mitochondrial fusion was inhibited in CK2α′^−/−^ cells, which was indicated by Mito-tracker staining. Arrows (red) indicate branched healthy mitochondria. Arrows (white) indicate spherical dysfunctional mitochondria (scale bar: 20 μm). **e** ECH recovered mitochondrial fusion induction in CK2α′^−/−^ lung fibroblasts which were transfected with CK2α′ plasmid. Arrows (red) indicate branched healthy mitochondria. Arrows (white) indicate spherical dysfunctional mitochondria (scale bar: 20 μm). **f** Mfn2 expression induced by ECH was downregulated in CK2α′ knock-down PC12 cells, which was analyzed by western blot. Data are expressed as the mean ± SD. ^##^*P* < 0.01 vs. control group, *P < 0.05, **P < 0.01 vs. OGD/R group. NS not significant

First, we knocked down CK2α′ with a specific siRNA to characterize the function of CK2 in cell viability (Supplementary Fig. 5a). We found that ECH increased PC12 cell viability under OGD/R conditions in negative control (NC) siRNA group but this effect was markedly reversed by CK2α′ siRNA treatment (Fig. 3a). We also verified the protective effect of ECH on primary cultured fibroblasts and found that ECH obviously increased wild-type CK2α′^+/+^ cell viability under OGD/R insult, while viability was significantly suppressed in CK2α′^−/−^ cells (Fig. 3b).

To explore the potential role of CK2α′ in mitochondrial fusion progression, Mito-tracker staining analysis was performed. The results revealed that ECH-mediated mitochondrial fusion was significantly blocked by CK2α′ siRNA (Fig. 3c, Supplementary Fig. 6a), indicating a crucial function of CK2α′ in mitochondrial fusion dynamics. The specificity of CK2α′ siRNA knockdown was investigated by western blot between CK2α′ and CK2α. The results showed that CK2α′ was obviously knocked down when CK2α′ siRNA was transfected into the cells. Although the expression of CK2α was also decreased to some degree, it was much less obvious as CK2α′, indicating that CK2α′ siRNA mainly targets CK2α′ but not CK2α (Supplementary Fig. 5a). Therefore, it can be concluded that ECH-mediated mitochondrial fusion was mainly achieved via targeting CK2α′ but not CK2α. Moreover, this observation was confirmed in CK2α′^−/−^ fibroblasts. As shown in Fig. 3d and Supplementary Fig. 6b, CK2α′ gene knockout significantly inhibited ECH-mediated mitochondrial fusion. In addition, transfection of CK2α′ plasmids, but not control vectors, into CK2α′^−/−^ cells phenocopied the mitochondrial fusion-promoting effect of ECH on CK2α′^+/+^ cells (Fig. 3e, Supplementary Fig. 6c). Furthermore, ECH was found to increase the expression of mitochondrial fusion protein Mfn2 with or without OGD/R insult; however, these effects were effectively reversed by CK2α′ siRNA treatment (Fig. 3f). Similar results were also observed at *Mfn2* gene transcription level (Supplementary Fig. 6d), indicating that CK2α′ plays an important role in mitochondrial fusion by promoting Mfn2 expression. Then, JC-1 staining was performed to investigate mitochondrial function and showed an obvious protective effect of ECH on MMP stability, which was antagonized by CK2α′ siRNA (Supplementary Fig. 6e). Mito-tracker staining assays showed that CK2α′ overexpression significantly promoted ECH-mediated mitochondrial fusion. However, CK2α overexpression did not play a significant role in promoting mitochondrial fusion upon ECH treatment. Moreover, our results showed that branched healthy mitochondria were markedly increased and spherical dysfunctional mitochondria were decreased upon CK2α′ overexpression but not for CK2α, particularly in OGD/R group (Supplementary Fig. 6f). Taken together, these observations suggest that CK2α′ is a crucial regulator that promotes mitochondrial fusion by inducing the expression of mitochondrial fusion gene *Mfn2*.

### CK2α′ binds to BTF3 as a direct substrate

**Fig. 4:**
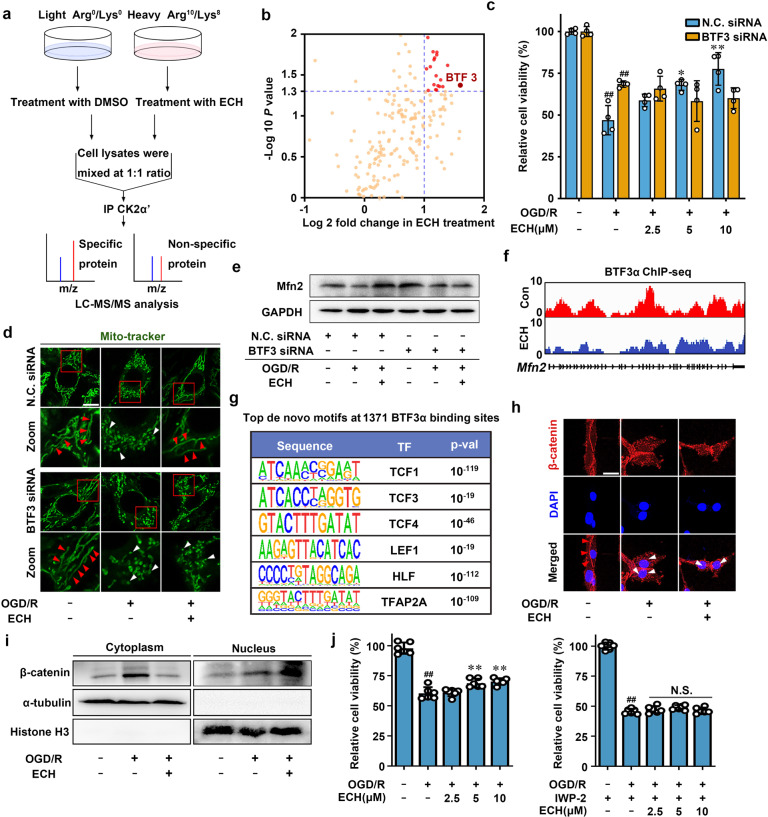
CK2α′ binds to basic transcription factor (BTF3) as a direct substrate. **a** Stable isotope labeling with amino acids in cell culture (SILAC)-coupled pull-down technology was used to identify the binding substrates of CK2α′. **b** Overview of the binding substrates of CK2α′ was identified by LC–MS/MS. **c** BTF3 knock-down inhibited ECH-mediated cell viability increase, which was detected by MTT assay. **d** ECH-induced mitochondrial fusion was inhibited in BTF3 knock-down cells, which was indicated by Mito-tracker staining. Arrows (red) indicate branched healthy mitochondria. Arrows (white) indicate spherical dysfunctional mitochondria (scale bar: 20 μm). **e** BTF3 knock-down inhibited Mfn2 expression, which was detected by western blot. **f** Genome browser view of BTF3α ChIP-seq signal on *Mfn2* gene loci. **g** Top enriched de novo transcription factor (TF) motifs within 14 bp upon ECH treatment. **h** Co-localization of β-catenin (red) and DAPI (blue) was detected by immunofluorescent staining. Arrows (red) indicate cytoplasmic location of β-catenin. Arrows (white) indicate nuclear translocation of β-catenin from the cytoplasm (scale bar: 20 μm). **i** ECH facilitated β-catenin nuclear translocation. **j** IWP-2 reversed ECH-dependent cell viability increase under OGD/R insult. Data are expressed as the mean ± SD. ^##^*P* < 0.01 vs. control group, *P < 0.05, **P < 0.01 vs. OGD/R group

We next sought to identify direct interaction proteins linking CK2 to mitochondrial fusion by pulldown assay in whole-cell extracts via stable isotope labeling with amino acids in cell culture (SILAC) (Fig. 4a). We captured several potential binding proteins using CK2α′ as a bait protein immobilized on beads. Among these proteins, BTF3 attracted our particular attention (Fig. 4b, Supplementary Table 4) because BTF3 is associated with mitochondrial function regulation and neuronal cell survival via gene transcriptional regulation.^28,29^ MTT assay showed that ECH increased cell viability in NC siRNA-transfected PC12 cells, and this effect was significantly reversed by BTF3 siRNA treatment (Fig. 4c, Supplementary Fig. 5b). Mito-tracker staining revealed that ECH-mediated mitochondrial fusion was blocked in BTF3 siRNA-transfected cells (Fig. 4d, Supplementary Fig. 7a), indicating a key role of BTF3 in the mitochondrial fusion process. This observation was further confirmed by western blotting and RT-PCR assays, which showed that ECH-dependent increases in Mfn2 protein and mRNA levels were significantly suppressed by BTF3 siRNA treatment (Fig. 4e, Supplementary Fig. 7b). Notably, immunofluorescence colocalization analysis revealed that ECH induced BTF3 cytoplasmic translocation to interact with CK2α′ (Supplementary Fig. 7c), suggesting that CK2α′ may act as a protein recruitment platform for BTF3 interaction.

BTF3 gene encodes two isoforms, BTF3α and BTF3β;^30^ thus, we further explored whether CK2α′ selectively interacts with either BTF3 isoform. His-tagged CK2α′ effectively captured both BTF3α and BTF3β; however, ECH promoted only the interaction of BTF3α with CK2α′. This observation was confirmed by pulldown analysis using BTF3α or BTF3β gene-overexpressing cells, indicating that CK2α′–BTF3α binding specifically responded to ECH treatment (Supplementary Fig. 7d). Then, we explored the potential phosphorylation sites on BTF3α to verify whether BTF3α is a direct phosphorylation substrate of CK2. As shown in Supplementary Fig. 7e, serine (S) 30 at the N-terminus of BTF3α was identified as a previously undisclosed phosphorylation site using LC–quadrupole mass spectrometry analysis upon ECH treatment. As shown in Supplementary Fig. 7f, ECH promotes *Mfn2* transcription upon BTF3α^WT^ overexpression compared to vector transfection. However, ECH could not induce obvious changes between vector and BTF3α^S30A^ transfection, indicating that BTF3α Ser30 play crucial roles for *Mfn2* transcription. BTF3α^S30A^ completely abolished BTF3α–CK2α′ interaction, indicating that Ser30 is essential for BTF3α–CK2α′ interaction (Supplementary Fig. 7g). Taken together, these observations suggest that S30 phosphorylation may be regulated by CK2.

### BTF3 promotes mitochondrial fusion in a Wnt/β-catenin-dependent manner

To confirm whether mitochondrial fusion is regulated by BTF3, we performed chromatin immunoprecipitation (ChIP) sequencing (ChIP-seq) analysis. As shown in Supplementary Fig. 8a, we identified 1229 high-confidence BTF3α-binding sites without ECH treatment and 1371 high-confidence BTF3α-binding sites with ECH treatment. Then, we used these high-confidence binding sites for further analyses. These binding sites were primarily found near gene-coding regions of the genome, including transcription start site (TSS)-downstream, exonic, intronic, intergenic, and TSS-upstream regions (Supplementary Fig. 8b). Notably, ChIP-seq analysis revealed that BTF3α accumulated at several mitochondrial dynamics-associated gene loci, such as *Mfn2* and *Fis1* loci (Fig. 4f, Supplementary Fig. 8c), indicating that mitochondrial fusion process could be mediated through the action of BTF3α on these genes.

**Fig. 5:**
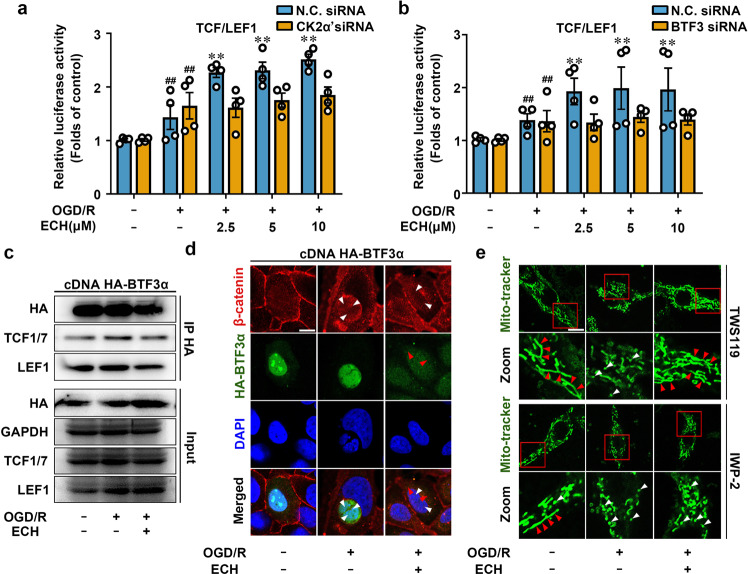
BTF3 promotes Mfn2 expression in Wnt/β-catenin signal-dependent manner. **a**, **b** ECH enhanced TCF/LEF1 reporter gene activity, which was inhibited by CK2α′ or BTF3 knock-down. **c** ECH inhibited the interaction of BTF3α with TCF1/7 and LEF1. HEK293T cells were transfected with HA-tagged BTF3α and treated by ECH (10 μM) for 6 h. Co-IP was performed with anti-HA antibody, followed by immunoblotting analysis. **d** Cytoplasmic translocation of HA-BTF3α (green) from nucleus and nuclear translocation of β-catenin (red) from cytoplasm were promoted by ECH (scale bar: 20 μm). **e** IWP-2 blocked ECH-induced mitochondrial fusion; however, TWS119 did not show similar effect. Arrows (red) indicate branched healthy mitochondria. Arrows (white) indicate spherical dysfunctional mitochondria (scale bar: 20 μm). Data are expressed as the mean ± SD. ^##^*P* < 0.01 vs. control group. *P < 0.05, **P < 0.01 vs. OGD/R group

Intriguingly, we also discovered several transcription factor-binding motifs from ChIP-seq data, including *TCF1*, *TCF3*, *TCF4*, *LEF1, HLF*, and *TFAP2A*, which were all highly associated with Wnt/β-catenin signaling (Fig. 4g).^31,32^ Thus, we hypothesized that BTF3α may interact with some master regulators in Wnt/β-catenin signaling pathway to modulate gene transcription in response to ECH treatment. To test this hypothesis, we first investigated whether ECH regulates the nuclear translocation of β-catenin, which is a hallmark of canonical Wnt signal activation. Our results showed that ECH markedly induced β-catenin nuclear translocation with or without OGD/R insult, suggesting that β-catenin is involved in ECH-mediated Wnt signal activation (Fig. 4h, i, Supplementary Fig. 8d). Next, TCF/LEF1 luciferase vector was used as Wnt pathway-responsive reporter to evaluate Wnt pathway activation at transcriptional level. Consistently, we found that ECH increased TCF/LEF1 luciferase activity in a concentration-dependent manner. However, ECH-mediated TCF/LEF1 luciferase activation was significantly inhibited by CK2α′ siRNA and BTF3 siRNA (Fig. 5a, b), suggesting a crucial function of CK2α′ and BTF3α in Wnt pathway activation. Additionally, Wnt inhibitor IWP-2 significantly blocked ECH-dependent TCF/LEF1 activation (Supplementary Fig. 8e) and reversed ECH-dependent increases in cell viability under OGD/R insult (Fig. 4j).

To further provide direct evidence that BTF3α is involved in Wnt/β-catenin pathway, we performed a coimmunoprecipitation (co-IP) assay. We found that ECH inhibited BTF3α binding to TCF1/7 and LEF1 under OGD/R conditions (Fig. 5c), suggesting that BTF3α directly interacts with Wnt/β-catenin signaling in OGD/R conditions. This observation was supported by immunofluorescence colocalization analysis, which showed that ECH promoted simultaneous BTF3α cytoplasmic translocation and β-catenin nuclear translocation (Fig. 5d). ChIP-PCR analysis revealed a significant enrichment of TCF1/7 at transcription factor-accessible regions of *Mfn2* upon ECH treatment (Supplementary Fig. 8f). This observation showed that TCF1/7 could directly bind to *Mfn2* promoter region. Thus, our findings are consistent with previous observation that TCF1/7 may bind to *Mfn2* promoter to activate *Mfn2* gene transcription and induce mitochondrial fusion (Supplementary Fig. 8g). We also used IWP-2 (Wnt inhibitor) and TWS119 (Wnt activator) to explore the potential effect of Wnt signaling on mitochondrial fusion. As shown in Fig. 5e and Supplementary Fig. 8h, IWP-2 significantly antagonized ECH-dependent mitochondrial fusion; however, TWS119 did not alter the effect of ECH on mitochondrial fusion, suggesting that ECH activates Wnt signaling similarly to TWS119. Taken together, these observations indicate that BTF3α facilitates mitochondrial fusion through a Wnt/β-catenin-dependent mechanism.

### CK2 exerts a neuroprotective effect by promoting mitochondrial fusion in an middle cerebral artery occlusion (MCAO) model

**Fig. 6:**
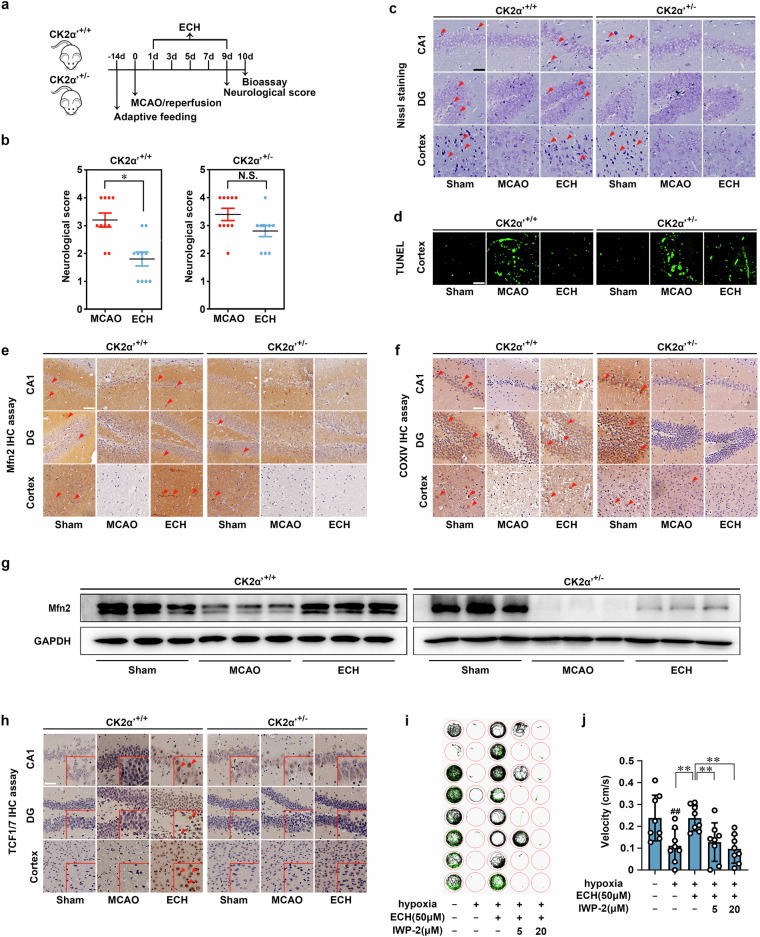
CK2 exerts neuroprotection via promoting mitochondrial fusion in MCAO model. **a** Scheme of neuroprotection evaluation of ECH in MCAO model. MCAO were established on mice and ECH (50 mg/kg) was administrated for every 2 days, followed by neurological score test in day 9 and bioassay in day 10. **b** ECH improved neurological score against MCAO insult in CK2α′^+/+^ mice, but showed no effect in CK2α′^+/−^ mice. **c** ECH protected neurons against MCAO insult in CK2α′^+/+^ mice, but showed no effect in CK2α′^+/−^ mice. Neuroprotection in hippocampal and cortical areas were detected by Nissl staining assay. Arrows indicate Nissl bodies (scale bar: 50 μm). **d** ECH inhibited cell apoptosis against MCAO insult in CK2α′^+/+^ mice, but showed no effect in CK2α′^+/−^ mice. Cell apoptosis was detected by TUNEL assay (scale bar: 50 µm). **e** Mfn2 expression was increased by ECH in CK2α′^+/+^ mice, but not in CK2α′^+/−^ mice (scale bar: 50 μm). f ECH-induced mitochondrial fusion was suppressed in CK2α′^+/−^ mice, which was stained with the specific mitochondrial marker COXIV (scale bar: 50 μm). **g** CK2α′^+/−^ blocked ECH-induced Mfn2 expression increase, which was detected by western blot assay. **h** TCF1/7 significantly translocated into nucleus upon ECH treatment (scale bar: 50 μm). **i**, **j** Swimming performance analysis for ECH and IWP-2 neuroprotection in ischemia/reperfusion-induced zebrafishes. Data are expressed as the mean ± SD. ^##^*P* < 0.01 vs. control group. *P < 0.05, **P < 0.01 vs. MCAO group. NS not significant

We next investigated whether CK2 is a therapeutic target for ischemic stroke in a mouse MCAO model (Fig. 6a). Because CK2α′ gene knockout leads to early embryonic lethality, we established CK2α′^+/−^ heterozygous C57/BL6 mice to test the neuroprotective effect of ECH. The results showed that MCAO wild-type (CK2α′^+/+^) mice showed obvious neurological deficits, such as hemianesthesia and hemiparesis, that were effectively relieved by ECH administration. However, we observed that ECH-mediated neurological recovery was significantly attenuated in CK2α′^+/−^ mice (Fig. 6b). To further investigate the role of CK2 in ECH-mediated effects on neuronal morphological features, we performed hematoxylin and eosin (HE) staining. MCAO induced obvious neuronal damage, including abnormal nuclei and neuronal shrinkage in the cerebral cortex and hippocampal regions. Treatment with ECH significantly reduced the numbers of shrunken cells in the cortex and hippocampal regions in CK2α′^+/+^ mice. However, this phenomenon did not occur in CK2α′^+/−^ mice (Supplementary Fig. 9a), suggesting a crucial role of CK2α′ in improving neurological functions. Then, TUNEL and Nissl staining assays were performed to evaluate neuronal apoptosis and neuronal morphology. As shown in Supplementary Fig. 9b, c, ECH exhibited a significant protective effect against neuronal apoptosis and promoted neuronal morphological reconstruction, but these effects were markedly attenuated in CK2α′^+/−^ mice.

To further delineate the effects of CK2α′ on mitochondrial functions in vivo, we examined the expression of mitochondrial fusion protein Mfn2 and mitochondrial marker cytochrome c oxidase subunit IV (COXIV). We observed that Mfn2 and COXIV expression was strongly induced by ECH treatment; however, this effect was disrupted in CK2α′^+/−^ mice (Fig. 6c, d), indicating that CK2α′ is a key contributing factor that facilitates Mfn2 activation and maintains mitochondrial morphology. We also confirmed this finding by analyzing Mfn2 protein expression in brain tissues using a western blot assay. As shown in Fig. 6e, ECH increased Mfn2 levels in CK2α′^+/+^ mice, but this effect was significantly blunted in CK2α′^+/−^ mice. Taken together, these findings indicate that CK2 is a crucial therapeutic target for ischemic cerebral injury and that CK2α′ can be targeted to promote mitochondrial fusion.

β-catenin acts as an intracellular signal transducer in transcriptional regulation and chromatin interactions. In addition, β‐catenin in the nucleus is mediated via T‐cell factor (TCF)/lymphoid enhancer-binding factor (LEF) transcription factors, which in association with β-catenin trigger Wnt-mediated transcription. Thus, TCF1/7 were determined by IHC staining as the major Wnt signaling players. Results showed that TCF1/7 significantly translocated into nucleus upon ECH treatment, suggesting that ECH activated Wnt signaling pathway in vivo (Fig. 6h). Moreover, zebrafish swimming performance analysis showed that ECH exhibited noticeable neuroprotective effects in hypoxia insult-induced zebrafish swimming behavioral injury model, which was significantly reversed by Wnt inhibitor IWP-2 (Fig. 6i, j). Collectively, these findings suggest that Wnt signaling is involved in ECH-mediated neuroprotective effect.

## Discussion

Mitochondrial fusion/fission dynamics widely influences neuronal functions, including neuronal survival and plasticity.^33,36^ Therefore, it is important to develop feasible strategies to regulate this process. Although accumulating evidence has revealed several proteins critical for the control of mitochondrial dynamics, such as Mfn1/2, Fis1, Mff, and Drp1,^34–36^ novel druggable targets that modulate this crucial biological process still urgently need to be identified. Our study reveals, for the first time, that CK2 is a fundamental molecular switch that promotes mitochondrial fusion process to provide neuroprotection against ischemia. Therefore, CK2 is a previously unrevealed therapeutic target for ischemic stroke that acts by controlling mitochondrial homeostasis.

Notably, we observed that CK2α′ subunit plays a crucial role in mitochondrial fusion by inducing BTF3α cytoplasmic translocation to form a CK2α′/BTF3α complex. We found this complex further promotes mitochondrial fusion by inducing Mfn2 transcription and expression through Wnt/β-catenin-dependent mechanism. Therefore, our findings provide a conceptual framework for pharmacologically targeting CK2 to facilitate mitochondrial fusion via a Wnt/β-catenin-related mechanism. Additionally, to the best of our knowledge, this is the first report of a covalent small-molecule regulator that targets CK2 to exert a neuroprotective effect. Our study thus reveals crucial information supporting future structure-based drug discovery for cerebral ischemia protection.

The currently available small-molecule regulators targeting CK2 family mainly bind to CK2 catalytic pocket.^37^ In this study, we identified an unexpected pharmacological binding domain containing K171 covalent site, which is far from the previously identified catalytic pocket. The three-dimensional structure of ECH-bound CK2α′ subunit showed a distinctive ligand-binding pocket containing R65, I95, E115, Q169, and G92 residues; this pocket provides a molecular basis for hydrogen bond formation between ECH and CK2α′. Moreover, such hydrogen bond formation may constitute a key precondition for further covalent binding of ECH to K171. Thus, the ligand-binding pocket may involve diverse intermolecular forces and guide novel CK2 small-molecule regulator discovery. In addition, ECH did not inhibit CK2 catalytic activity at classic phosphorylation motif but rather regulated a previously undiscovered phosphorylation site on BTF3. We propose that ECH may exert this effect on CK2α′ via a dynamic allosteric mechanism that enables selectivity for different target motifs. Further studies will be required to understand the precise mechanism by which ECH regulates CK2 with substrate specificity.

Wnt/β-catenin signaling is highly involved in cell survival.^38–40^ In the presence of ligands, Wnt signal activation induces the accumulation of β-catenin through a destructive complex of casein kinase 1 (CK1), APC, Axin, and GSK3β, further stimulating β-catenin nuclear translocation and Wnt target gene transcription.^41–43^ CK2 has been previously reported to phosphorylate β-catenin at Thr393,^44^ which increases β-catenin expression via ubiquitin–proteasome system and activates Wnt signaling pathway.^45^ Intriguingly, our current study reveals a previously unknown mechanism of CK2-dependent Wnt/β-catenin signal activation, which depends on transcription factor BTF3 as a scaffold protein. For the first time, we report that CK2α′/BTF3α complex promotes β-catenin nuclear translocation and subsequent transcriptional activation of mitochondrial fusion gene *Mfn2*. We speculate that ECH may enable a CK2α′ conformational change to promote BTF3α-binding domain exposure and recruit BTF3α in the cytoplasm. In addition, we hypothesized that the co-localization may be attributed to ECH-dependent conformational regulation of CK2α′, which further induces BTF3 recruitment to cytoplasm. BTF3 translocation allows easier access of Wnt-related transcription factors to their target loci, such as TCF/LEF1. Thus, CK2α′ may serve as a scaffold or “sponge” for inactivating BTF3 transcriptional functions.

Interestingly, BTF3 has long been considered to be a general transcription factor for gene transcriptional regulation.^46^ However, we discovered that BTF3 works as an adaptor by interacting with CK2α′ to form a protein–protein binary complex in the cytoplasm. Thus, our observation indicates that BTF3 plays diverse biological roles and expands our understanding of general transcription factor functions. Importantly, in canonical Wnt/β-catenin pathway, CK1 is involved in regulating β-catenin activation;^47,48^ however, the biological function of CK2 in Wnt/β-catenin signaling is largely unexplored. Our findings indicate that CK2 also contributes to β-catenin activation, revealing a previously undiscovered mechanism of Wnt/β-catenin signaling regulation. Moreover, CK2 has been found to be overexpressed in several human tumors such as malignant peripheral nerve sheath tumors, acute myeloid leukemia, and human prostate cancer.^49^ In particular, recent data has shown that CK2 and β-catenin can be co-precipitated with Wnt signaling intermediate Dvl, suggesting a crucial role of CK2 in Wnt-driven cancer progression.^50^ Thus, CK2 seems to be a promising therapeutic target in cancers. However, the potential function of CK2-mediated Wnt/β-catenin signal in neuroprotection during cerebral ischemia still remains unclear. In our research, ECH has been discovered to activate Wnt signaling through CK2/BTF3α to exert neuroprotection, which indicates a previously unknown function of CK2/Wnt signal axis in treatment of cerebral ischemia.

Collectively, our findings provide, for the first time, a proof of concept that CK2 is a promising druggable target that promotes mitochondrial fusion via a Wnt/β-catenin-dependent mechanism. In addition, our study demonstrates the feasibility of using the small molecule ECH as a novel molecular template to target CK2 for future clinical therapies for ischemic stroke.

## Materials and methods

### Chemicals and reagents

ECH (C_35_H_46_O_20_) was purchased from Shengshi Technology (Baoji, Shanxi, China). MTT and DAPI were purchased from Sigma (St. Louis, MO, USA). l-glutathione (reduced) was obtained from Solarbio Science & Technology (Beijing, China). Protein A/G-agarose was obtained from Biogot Technology (Nanjing, Jiangsu, China). Lipofectamine RNAiMAX and Lipofectamine 2000 were obtained from Thermo (Waltham, MA, USA). Pronase was obtained from Roche Diagnostics GmbH (Mannheim, Germany). Human recombinant CK2α′ protein was purchased from Sino Biological (Beijing, China). An LDH Cytotoxicity Detection Kit, a JC-1 assay kit, Lyso-tracker, Golgi-tracker, and Mito-tracker were purchased from Beyotime Biotechnology (Nanjing, Jiangsu, China). ER-tracker and Super ECL Detection Reagents were purchased from Yeasen (Shanghai, China). TWS119 was obtained from Selleck Chemicals (Houston, TX, USA). IWP-2 was obtained from Bide Medical Technology (Shanghai, China). Primary antibodies and secondary antibodies were obtained from Cell Signaling Technology (Beverly, MA, USA) and Abcam (Cambridge, UK). A SuperLight Dual Luciferase Reporter Gene Assay Kit was obtained from Lablead (Beijing, China). A StarPure Endo-free Plasmid Maxiprep Spin Kit was purchased from Genstar Biotechnology (Beijing, China). SiRNA was obtained from GenePharma (Suzhou, Jiangsu, China). Recombinant plasmids were constructed by Generay Biotech (Shanghai, China).

### Cell culture

Rat pheochromocytoma (PC12) and human embryonic kidney 293T (HEK293T) cells were obtained from the Peking Union Medical College Cell Bank (Beijing, China). The cells were cultured in a cell incubator at 37 °C under 5% CO_2_. The medium was high-glucose DMEM with 10% FBS, penicillin (100 U/ml) and streptomycin (100 μg/ml).

### OGD/R model

Cells were cultured with DMEM for 12 h to induce adherence, and then the DMEM was replaced with EBSS. The cells were placed in a sealed incubator (C-31, Mitsubishi, Nagasaki, Japan) with a Bio-Bag (C-1, Japan) for 4 h. Then, the EBSS was replaced with DMEM containing different concentrations of ECH (0, 2.5, 5, 10 μM), and the cells were cultured under normal culture conditions for 24 h.

### Cell viability analysis

To test cell viability, cells were first inoculated into 96-well plates. After treatment, the culture medium was removed, and MTT working solution (0.5 mg/ml) was added to the 96-well plates (100 μl/well). Formazan formed after 4 h at 37 °C. Then, the formazan was solubilized in DMSO, and the optical density (OD) was detected at 570 nm by a microplate reader (Austria GmbH 5082 Grodlg, Tecan, Männedorf, Switzerland).

### LDH analysis

LDH analysis was performed in accordance with the instructions of an LDH kit (Beyotime Biotechnology). After treatment, the medium from each well was collected and centrifuged at 13,000 rpm for 5 min. Then, 60 μl of LDH detection solution was mixed with 120 μl of supernatant from each well, and the mixture was incubated at 37 °C in the dark for 30 min. The OD was measured at 490 nm by a microplate reader (Tecan).

### MMP analysis

After treatment, cells were treated with JC-1 working solution (10 μg/ml) and incubated at 37 °C in a 5% CO_2_ incubator for 30 min. The JC-1-treated cells were washed with PBS three times for 2 min each time, and images were obtained with a fluorescence microscope (IX73, Olympus, Tokyo, Japan) at 514 nm (excitation) and 590 nm (emission).

### Mito-tracker staining assay

Cells were cultured in confocal dishes for 24 h. After treatment, Mito-tracker solution (200 nM) was added to the cells, and the cells were incubated at 37 °C in a 5% CO_2_ incubator for 40–60 min. Images were obtained by fluorescence microscopy (LSM880, Zeiss, Oberkochen, Germany) at 490 nm (excitation) and 516 nm (emission).

### TEM analysis

Cells were collected and placed in 2% glutaraldehyde at 4 °C for 8 h. The fixing solution was removed, 1% osmic acid was added, and the cells were incubated at 4 °C for 2 h. The cells were then dehydrated with gradient acetone solutions, soaked, embedded, polymerized with Epon 812 epoxy resin, and sliced with an ultrathin slicer (0.5 μm) (EM UC7, Leica, Wetzlar, Germany). Then, 0.25% lead citrate and 5% uranium dioxide-acetate were added, and the cells were incubated at 25 °C for 2 min. After PBS washes were performed, TEM (HT7700, Hitachi, Tokyo, Japan) was used for observation. Images were randomly obtained for each group from more than five fields.

### Western blotting

Cells were collected at 3000 rpm for 3 min after experimental treatment. RIPA lysis buffer with protease inhibitors was used to lyse the cells on ice for 30 min. The cell supernatant was collected for 8–15% SDS–PAGE separation, and the proteins were electrotransferred onto PVDF membranes. Then, the PVDF membranes were incubated with primary antibodies overnight at 4 °C and with HRP-conjugated secondary antibodies for 1 h at room temperature. The membranes were developed with Super ECL Detection Reagent and scanned with a Tanon 5200 Imaging Analysis System (Tanon, Shanghai, China).

### ECH cellular target identification

The cellular targets of ECH were determined in a pulldown experiment according to previously published methods, with some modifications.^30^ Cell supernatant was mixed with vehicle or ECH beads and then incubated at 4 °C overnight. The targeted proteins were captured by the ECH beads. Subsequently, the proteins were denatured by SDS, separated by 10% SDS–PAGE and visualized by silver staining. The targeted proteins were detected by LC–MS/MS analysis using a Nano-HPLC-tandem LTQ-Orbitrap Velos Pro mass spectrometer (LTQ-Orbitrap, Thermo, Waltham, MA, USA).^31^

### SPR analysis

The biomolecular interaction between ECH and CK2α′ was analyzed by SPR using a Biacore T200 system (GE Healthcare, Uppsala, Sweden) at 25 °C. First, a CM5 sensor chip was activated by using N-hydroxysulfosuccinimide (sulfo-NHS)/1-ethyl-3-(3-dimethylaminopropyl)carbodiimide (EDC). After that, the activated CM5 chip was coupled with recombinant human CK2α′ protein. During coupling, acetic acid (500 μg/ml, pH 4.0) was used to dilute the CK2α′ protein, and blocking was then performed with ethanolamine. ECH was dissolved in PBS+Tween 20 (PBST); the final concentration of ECH was 50 μM at the time of the SPR test. The results were analyzed with Biacore evaluation software (T200 Version 1.0).

### ITC analysis

The affinities and thermodynamics of CK2α′ protein binding with ECH were determined using a MicroCal PEAQ-ITC (Malvern Instruments, Malvern, UK). In ITC analysis, 30 μM CK2α′ protein was prepared in PBS (pH 7.4). ECH was dissolved in the same buffer as the CK2α′ protein (PBS, pH 7.4). The sample well (*V* = 280 µl) was prefilled with 30 μM CK2α′ protein, and ECH (0.5 mM) was preinjected into the instrument. During titration, an initial injection of ECH (0.4 µl) was followed by 29 successive injections of 1.2 µl with a 150 s interval.

### Chromatin immunoprecipitation

A mixture of Lipofectamine 2000 and constructed HA-BTF3α plasmid (GeneRay, Beijing, China) was added to HEK293T cells in Opti-MEM culture medium. After 6 h, the Opti-MEM was replaced with DMEM. Forty-eight hours later, the plasmid was expressed successfully and could be used for further experiments. The cells were divided into control and ECH groups. The cells in the ECH group were treated with ECH (10 μM) for 6 h. The proteins in the supernatant were crosslinked with 1% formaldehyde for 10 min in PBS, and the reaction was terminated with 125 mM glycine for 5 min. DNA was extracted by using a Pierce Magnetic ChIP Kit. DNA quality was evaluated with an Agilent Bioanalyzer 2100 system (Agilent Technologies, Palo Alto, CA, USA). Subsequently, the DNA fragments were sequenced on an Illumina HiSeq platform.

### Plasmid construction

Human CK2α′, BTF3α, and BTF3β cDNA sequences were cloned into a pCMV-HA vector containing an HA tag sequence at the N-terminal region. Moreover, human CK2α′, CK2α, and CK2β were cloned into a pGEX-4T-1 vector containing a GST tag sequence at the N-terminal region. All sequences were obtained from NCBI.

### In vitro primary lung fibrolast (LF) culture

Primary LFs were isolated from CK2α′^−/−^ mice. Lung tissues were isolated, transferred to cell culture dishes and cut to a size of 1 mm^3^. The medium was high-glucose DMEM with 10% FBS, penicillin (100 U/ml), and streptomycin (100 μg/ml). After the primary LFs were allowed to attach for 72 h, the lung tissues were removed. Cell passage was performed every 3 days. LFs were cultured in a cell incubator at 37 °C and 5% CO_2_ for further experiments.

### Transient transfection of plasmids and siRNA

A mixture of Lipofectamine RNAimax and siRNA (GenePharma, Suzhou, Jiangsu, China) was added to HEK293T cells in Opti-MEM. The medium was changed to DMEM after 4–6 h of culture, and the cells were incubated at 37 °C in a 5% CO_2_ incubator. Forty-eight hours later, the siRNA was expressed successfully and could be used for further experiments. The siRNAs used are listed in Supplementary Table S1.

The constructed plasmids (GeneRay, Beijing, China) were premixed with Lipofectamine 2000 in Opti-MEM (Thermo) and then applied to the cells. The medium was changed to DMEM after 4–6 h. The cells were incubated at 37 °C in a 5% CO_2_ incubator for 48 h prior to further experiments.

### Immunofluorescence analysis

Cells were transiently transfected with the indicated plasmids for 48 h. After experimental treatment, the cells were fixed in 4% paraformaldehyde for 30 min and then permeabilized with 0.5% Triton X-100 (in PBS) for 30 min at room temperature. After washing the permeabilized cells three times with PBS, 5% BSA (in PBST) was added to the cells for 30 min. After that, the cells were incubated with primary antibodies overnight at 4 °C. Subsequently, the cells were incubated with a rabbit Dylight-594-conjugated or mouse Alexa Fluor 488-conjugated secondary antibody for 1 h at room temperature. Finally, the cells were incubated with DAPI (20 μg/ml). Images were obtained (594/618 nm for the rabbit Dylight-594-conjugated antibody; 488/519 nm for the mouse Alexa Fluor 488-conjugated antibody) using fluorescence microscopy (Zeiss).

### TCF/LEF1 luciferase reporter gene assay

HEK293T cells were transiently transfected with a mammalian luciferase reporter vector of the *Homo sapiens* TCF/LEF1 response element with Lipofectamine 2000 for 48 h. An OGD/R model was established and treated with ECH for 24 h. After that, the cells were lysed, and a dual luciferase reporter gene assay (Bioassays, Hayward, CA, USA) was used to detect TCF/LEF1 reporter gene luciferase activity with a fluorescence spectrophotometer (PerkinElmer, Waltham, MA, USA).

### Experimental animals

CK2α′^+/−^ mice were generated by disrupting CK2α′ gene expression via homologous recombination in a C57BL/6 background. CK2α′^+/−^ mice were generated using the CRISPR/Cas9 system (Cyagen Biosciences, Guangzhou, China). The genotyping primers for CK2α′^+/−^ were as follows: forward, 5-AGATGGCCACAATGATTCGC-3′; and reverse, 5′-TCTCACCATCCTCCCAACTC-3′. During the experiment, all mice were kept under a 12 h/12 h light/dark cycle. All mice had access to water and food ad libitum throughout the study period. The procedures for daily animal care and experiments were approved by the Institutional Animal Care and Use Committee of Peking University (License no. LA2017011).

### Establishment of the MCAO model

An MCAO model was established according to the Longa line embolism method with a minor modification. Briefly, the external carotid artery (ECA), common carotid artery (CCA), internal carotid artery (ICA), and pterygopalatine artery (PPA) were separated, and a filament was threaded into the ICA until the blood supply of the middle cerebral artery was blocked. Then, 1.5 h after occlusion, the filament was removed to induce reperfusion. The body temperature was kept at ~37 °C. The sham group was treated similarly to the MCAO group except that no filament was inserted.

### Neurological-deficit evaluation

The Zea-Longa scoring system (scaled from 0 to 4) was used to estimate the degree of cerebral injury caused by ischemic insult. The neurological score was evaluated after 9 days of ECH (50 mg/kg) administration. The neurological scoring system was as follows: 0, exhibits no obvious neurological deficits; 1, unable to extend the right foreleg normally; 2, walks in circles; 3, exhibits walking imbalance; 4, exhibits numbness and cannot walk.

### Zebrafish swimming performance analysis

Water was filled with high-purity nitrogen until the dissolved oxygen in the water was below 0.3 mg/L. Anoxic environment was simulated and 5 dpf juvenile fish was added. The juvenile fish sank to the bottom and stood still for 3 min as anoxic end point. Fish were taken out and randomly divided into four groups with 10 fishes in each group. Eight of them were randomly selected from each group, and moved into 48-well plates. The swimming track and data were obtained by zebra fish behavior detection system (Zebralab, Viewpoint, Lyon, France).

### Statistical analysis

The results are shown as the means ± SDs. GraphPad Prism 5.0 software was used to carry out one-way analysis of variance (ANOVA) with the least significant difference post hoc test to analyze the data. *P* < 0.05 was considered to indicate statistical significance.

## Supplementary information

Supporting information
