# Correction: Small molecule induces mitochondrial fusion for neuroprotection via targeting CK2 without affecting its conventional kinase activity

**DOI:** 10.1038/s41392-021-00533-3

**Published:** 2021-03-12

**Authors:** Ke-Wu Zeng, Jing-Kang Wang, Li-Chao Wang, Qiang Guo, Ting-Ting Liu, Fu-Jiang Wang, Na Feng, Xiao-Wen Zhang, Li-Xi Liao, Mei-Mei Zhao, Dan Liu, Yong Jiang, Pengfei Tu

**Affiliations:** 1 State Key Laboratory of Natural and Biomimetic Drugs, School of Pharmaceutical Sciences, Peking University, Beijing, 100191 China; 2 Proteomics Laboratory, Medical and Healthy Analytical Center, Peking University Health Science Center, Beijing, 100191 China

**Keywords:** Target identification, Target identification

Correction to: *Signal Transduction and Targeted Therapy* 10.1038/s41392-020-00447-6, published online 19 February 2021

In the process of collating the published data, the editors noticed one inadvertent mistake occurred during the production process in Fig. 6 that needs to be corrected.^1^ The correct data are provided as follows. The key findings of the article are not affected by these corrections.

**Fig. 6:**
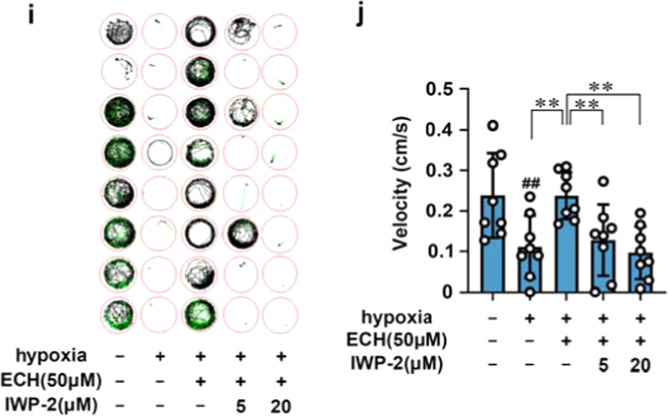
**i**, **j** Swimming performance analysis for ECH and IWP-2 neuroprotection in ischemia/reperfusion-induced zebrafishes. Data are expressed as the mean ± SD. ^##^*P* < 0.01 vs control group. *P < 0.05, **P < 0.01 vs MCAO group. NS not significant

The original article has been corrected.
