# Correction: Small molecule induces mitochondrial fusion for neuroprotection via targeting CK2 without affecting its conventional kinase activity

**DOI:** 10.1038/s41392-025-02218-7

**Published:** 2025-04-04

**Authors:** Ke-Wu Zeng, Jing-Kang Wang, Li-Chao Wang, Qiang Guo, Ting-Ting Liu, Fu-Jiang Wang, Na Feng, Xiao-Wen Zhang, Li-Xi Liao, Mei-Mei Zhao, Dan Liu, Yong Jiang, Pengfei Tu

**Affiliations:** 1 State Key Laboratory of Natural and Biomimetic Drugs, School of Pharmaceutical Sciences, Peking University, Beijing 100191, China; 2 Proteomics Laboratory, Medical and Healthy Analytical Center, Peking University Health Science Center, Beijing 100191, China

**Keywords:** Target identification, Target identification

Correction to: *Signal Transduction and Targeted Therapy* 10.1038/s41392-020-00447-6, published online 19 February 2021

During the collation of raw data, the authors identified an inadvertent duplication error in Figure 6c, which required correction following the article’s online publication.^1^ Specifically, an oversight during image processing resulted in the unintended reuse of the same dentate gyrus (DG) region image for the CK2α′+/+ + ECH treatment group and the CK2α′+/- + ECH treatment group. In the Nissl staining panel, the image representing the dentate gyrus (DG) region for the CK2α+/+ + ECH treatment group was unintentionally replaced with an incorrect image in the original version. This error has now been corrected, and the updated figure accurately reflects the intended data for this experimental group. Importantly, this correction does not affect the statistical analyses, conclusions, or overall integrity of the study. The revised figure is provided below.

Incorrect Figure 6c
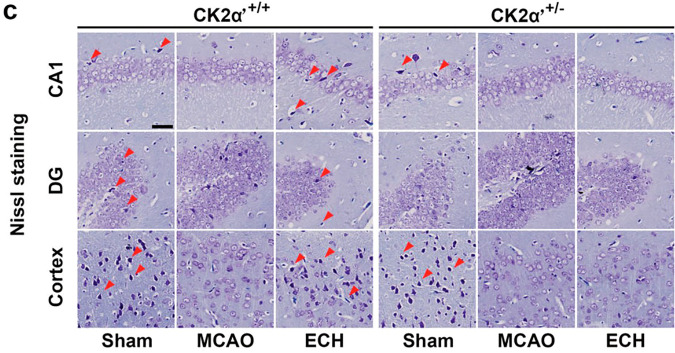


The revised Figure 6c
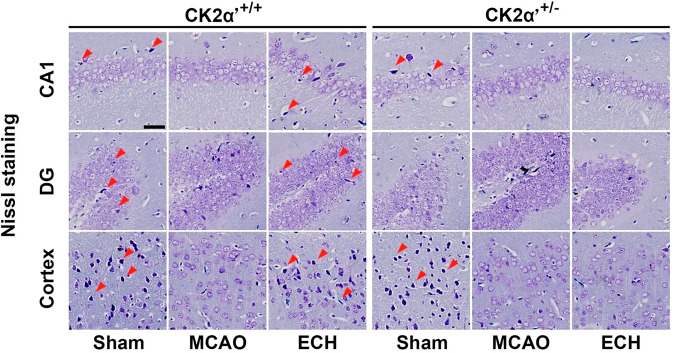


The original article has been corrected.
