# Supplementary material for: Small molecule induces mitochondrial fusion for neuroprotection via targeting CK2 without affecting its conventional kinase activity
Source: Signal Transduct Target Ther. 2021 Feb 19;6:71. doi: 10.1038/s41392-020-00447-6 (PMC7893052; doi:10.1038/s41392-020-00447-6)
Supplement: Supplementary file 1 — Supporting information [file 41392_2020_447_MOESM1_ESM.doc]

Supplementary Materials for

**Small molecule induces mitochondrial fusion for neuroprotection via targeting CK2 without affecting its conventional kinase activity**

**Running title: mitochondrial fusion induction via CK2**

Ke-Wu Zeng^1#^, Jing-Kang Wang^1#^, Li-Chao Wang^1^, Qiang Guo^1^, Ting-Ting Liu^1^, Fu-Jiang Wang^1^, Na Feng^1^, Xiao-Wen Zhang^1^, Li-Xi Liao^1^, Mei-Mei Zhao^1^, Dan Liu^2^, Yong Jiang^1^ & Pengfei Tu^1^*

^1^ State Key Laboratory of Natural and Biomimetic Drugs, School of Pharmaceutical Sciences, Peking University, Beijing 100191, China.

^2^ Proteomics Laboratory, Medical and Healthy Analytical Center, Peking University Health Science Center, Beijing 100191, China.

*Corresponding author. e-mail: pengfeitu@ bjmu.edu.cn (PF Tu)

# These authors contributed equally to this work.

This WORD file includes:

Figures. S1 to S9

Supplementary tables for methods

**Figure. S1**


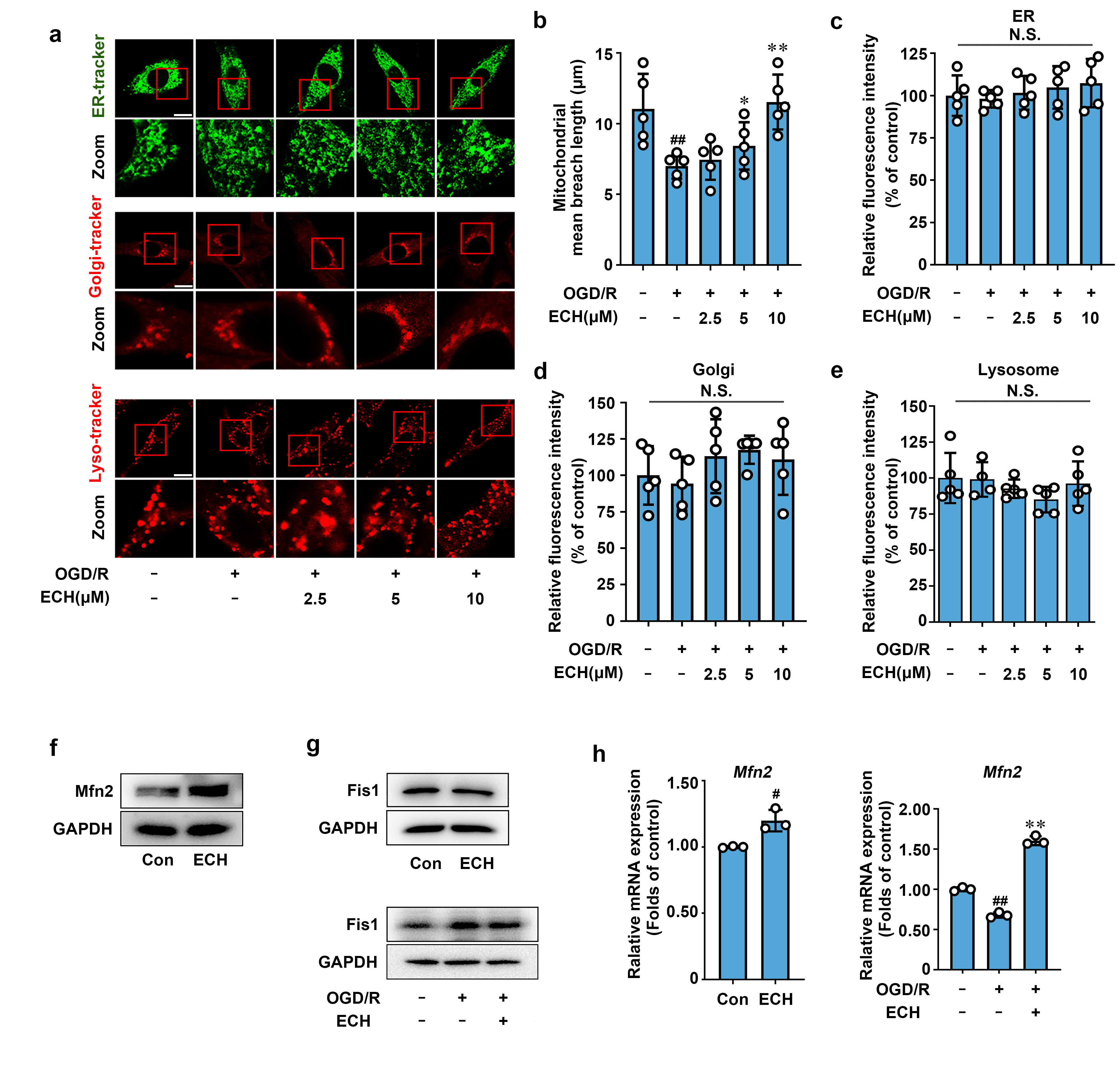


**Figure S1.** **ECH promotes mitochondrial fusion process for neuroprotection by inducing Mfn2 expression. a** ECH showed no effect on endoplasmic reticulum (ER), [Golgi](../../../../C:/Program%20Files%20(x86)/Youdao/Dict/8.7.0.0/resultui/html/index.html#/javascript:;) [apparatus](../../../../C:/Program%20Files%20(x86)/Youdao/Dict/8.7.0.0/resultui/html/index.html#/javascript:;) and lysosomes against OGD/R insult in PC12 cells. **b** Mitochondrial mean breach length (μm) quantitative analysis of Fig. 1c was performed using ImageJ. **c-e** Relative fluorescence intensities (ER-tracker, Golgi-tracker, Lyso-tracker) of Fig. S1a were performed using ImageJ. **f** ECH (10 μM) increased Mfn2 protein expression without OGD/R insult, which was analyzed by western blot. **g** ECH (10 μM) had no effect on Fis1 expression which was analyzed by western blot. **h** ECH (10 μM) induced *Mfn*2 gene transcription, which was analyzed by RT-PCR analysis. Data are expressed as the mean ± SD. ^#^*P* < 0.05, ^##^*P* < 0.01 vs control group, ***P* < 0.01 vs OGD/R group.

**Figure. S2**

**
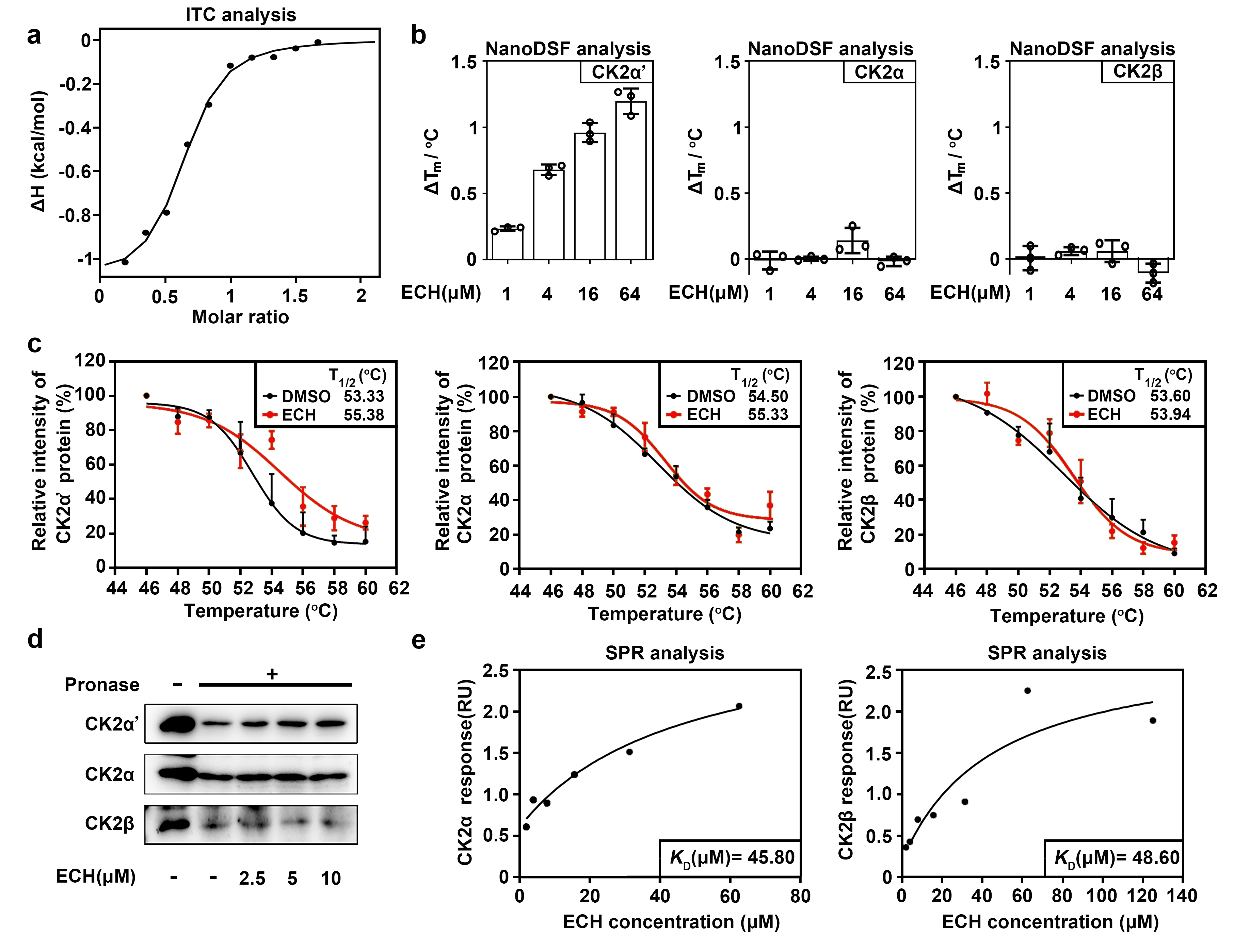
**

**Figure S2. ECH selectively interacted with CK2α’. a** ECH potently bound to CK2α’, which was detected by ITC analysis. **b** ECH improved the midpoint temperature (T_m_) of CK2α’ but had no effect on CK2α and CK2β, which was analyzed by NanoDSF assay. **c** ECH efficiently protected CK2α’ from temperature-dependent protein degradation in PC12 cells but had no effect on CK2α and CK2β, which was measured by CETSA assay. **d** ECH improved the resistance of CK2α’ to proteases but had no effect on CK2α and CK2β. **e** ECH showed weak interaction with CK2α and CK2β.

**Figure. S3**


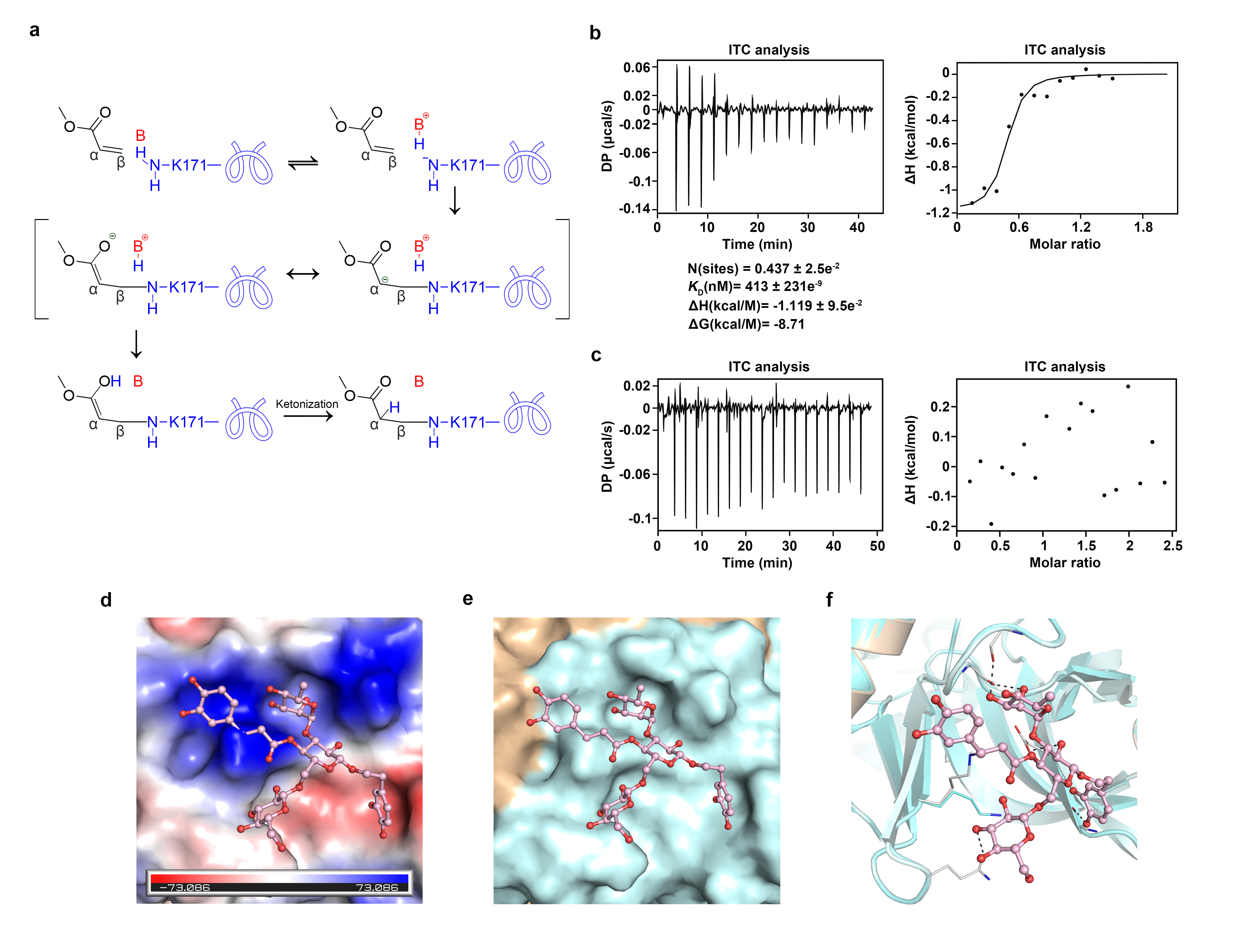


**Figure S3.** **The proposed covalent binding mode of ECH with CK2α’. a** ECH directly targets the Lys171 of CK2α’ via the Michael addition of amino lysine to α, β-unsaturated carbonyl. **b** Calorimetric titration of ECH with CK2α’ (R173A). **c** Calorimetric titration of ECH with CK2α’ (K171A). **d** Electrostatic surface representation of ECH binding site. ECH is shown in ball-and-stick model. **e** Surface representation of ECH-binding cavity. **f** Superposition of CK2α’-ECH (bluewhite) with PDB 5OOI (cyan). ECH is shown in ball-and-stick model and residues are shown in lines.

**Figure. S4**

**
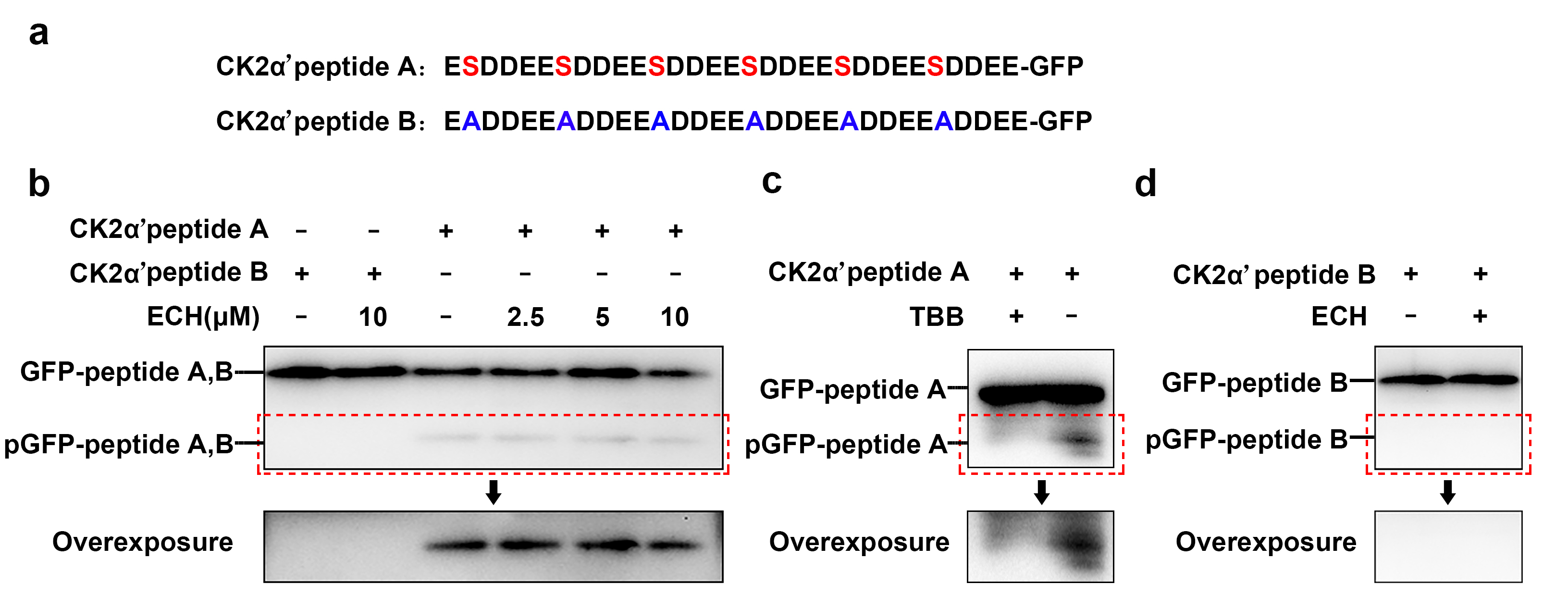
**

**Figure S4.** **ECH did not affect the serine site on classical CK2α’ phosphorylation motif. a** The amino acid sequence of classical phosphorylation motif CK2α’ peptide A and negative control peptide B. Serine (red) (Ser 2, 7, 12, 17, 22) in CK2α’ peptide A was the CK2α’ classical phosphorylation site. Alanine (blue) (Ala 2, 7, 12, 17, 22) in CK2α’ peptide B was the negative control site. **b** ECH did not affect the serine site on CK2α’ peptide A, which was detected by Native-PAGE and incubated with anti-GFP antibody. **c** TBB (CK2α’ inhibitor, 10 μM) blocked CK2α’ activity. **d** ECH did not [phosphorylate](../../../../C:/Program%20Files%20(x86)/Youdao/Dict/8.7.0.0/resultui/html/index.html#/javascript:;) negative control sequence CK2α’ peptide B.

**Figure. S5**


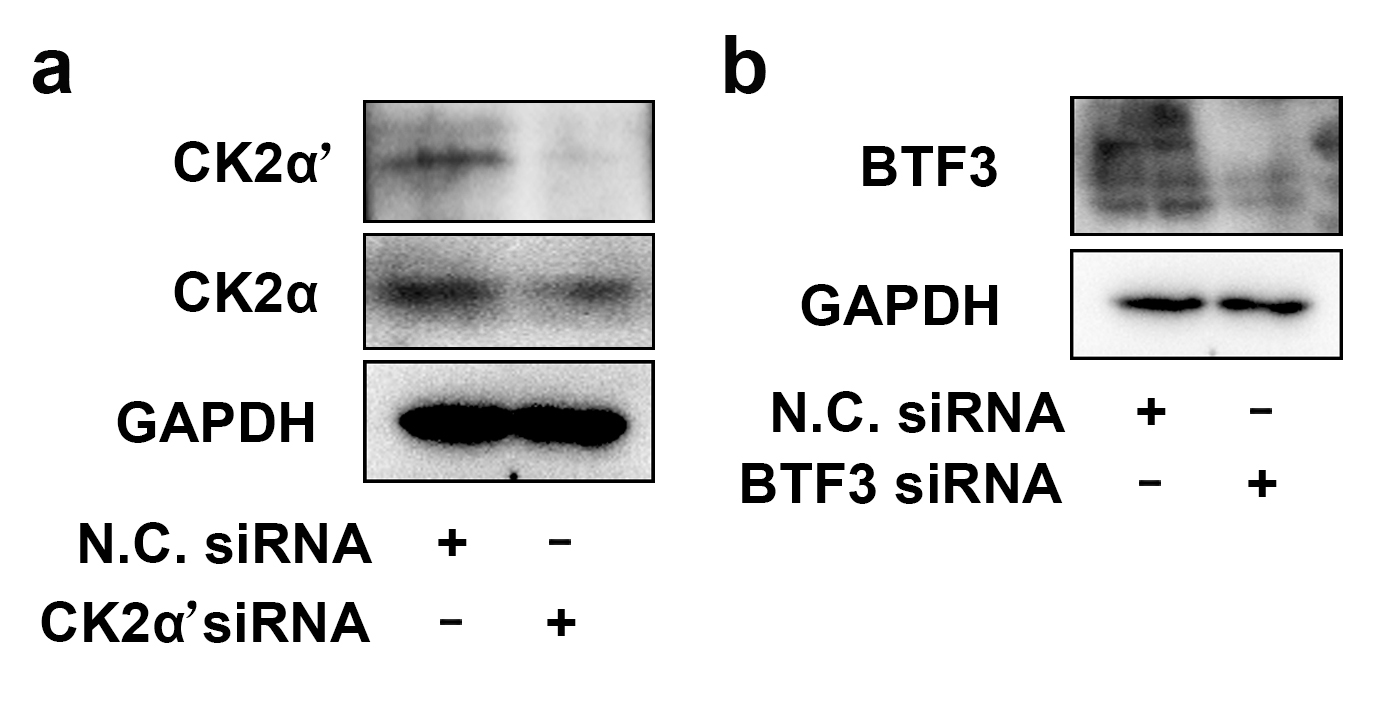


**Figure S5. siRNA knock-down efficiency verification. a** CK2α’ siRNA reduced CK2α’ expression in PC12 cells. **b** BTF3 siRNA reduced BTF3 expression in PC12 cells.

**Figure. S6**

**
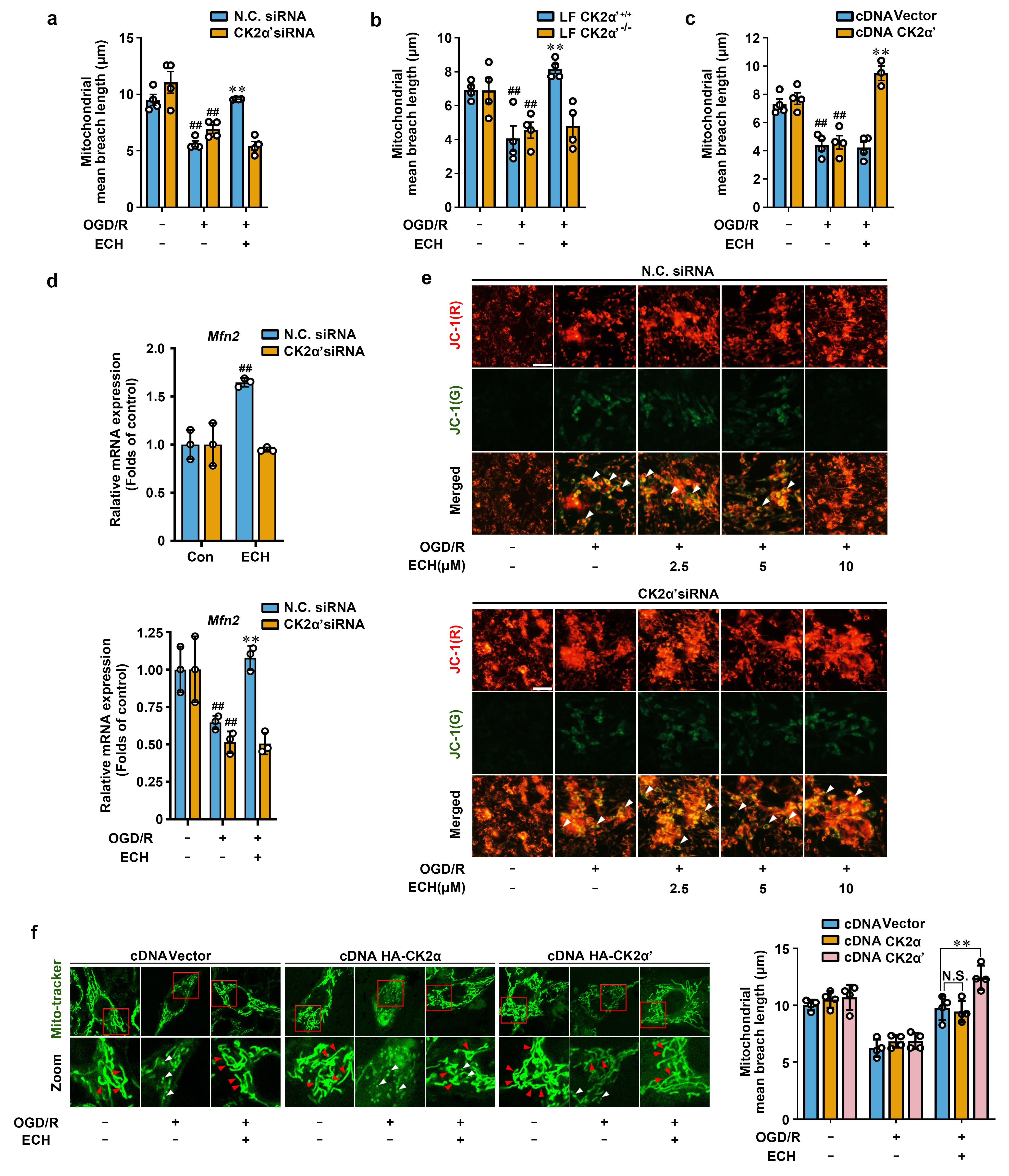
**

**Figure S6. CK2α’ plays a fundamental role in modulating mitochondrial function. a-c** Mitochondrial mean breach length (μm) quantitative analysis of Fig. 3c-e was performed using ImageJ. **d** *Mfn*2 transcription induced by ECH was downregulated in CK2α’ knock-down PC12 cells, which was analyzed by RT-PCR. **e** Knock-down of CK2α’ blocked ECH-mediated mitochondrial membrane potential increase, which was detected by JC-1 staining assay. Arrows indicate depolarized mitochondria (scale bar: 100 μm). **f** CK2α’ overexpression significantly promoted ECH-mediated mitochondrial fusion. Arrows (red) indicate branched healthy mitochondria. Arrows (white) indicate spherical dysfunctional mitochondria (scale bar: 20 μm). Data are expressed as the mean ± SD. ^##^*P* < 0.01 vs control group, ***P* < 0.01 vs OGD/R group.

**Figure. S7**


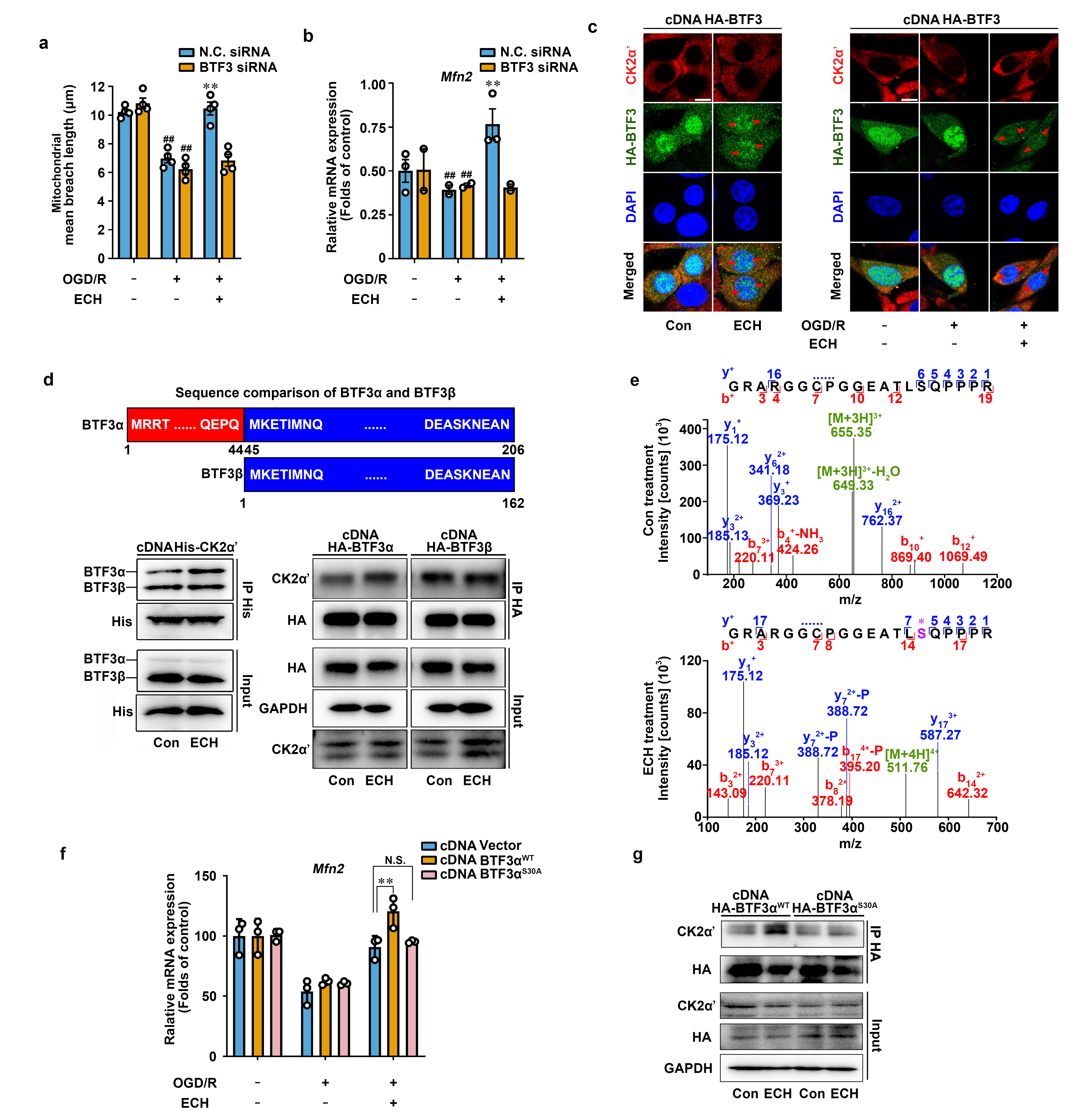


**Figure S7. CK2α’ binds to basic transcription factor (BTF3) as a direct substrate.**  **a** Mitochondrial mean breach length (μm) quantitative analysis of Fig. 4d was performed using ImageJ. **b** *Mfn*2 transcription induced by ECH was downregulated in BTF3 knock-down cells, which was analyzed by RT-PCR. **c** Co-localization of HA-tagged BTF3 (green) with CK2α’ (red). Arrows indicate cytoplasmic translocation of BTF3 from nucleus (scale bar: 20 μm). **d** BTF3α was identified as the binding substrate of CK2α’. HEK293T cells were transfected with His-tagged CK2α’ or HA-tagged BTF3α/BTF3β plasmids. Co-IP was performed with indicated antibodies followed by immunoblotting analysis. **e** Serine30 (S30) in BTF3α was identified as an undisclosed phosphorylation site. PC12 cells were incubated with ECH (10 μM) for 6 h, then cell lysate was detected by LC-MS/MS analysis. **f** Serine 30 (S30) on BTF3α is crucial for ECH-dependent *Mfn2* transcription. **g** BTF3α^S30A^ abolished BTF3α-CK2α' interaction. Data are expressed as the mean ± SD. ^##^*P* < 0.01 vs control group, ***P* < 0.01 vs OGD/R group.

**Figure. S8**

**
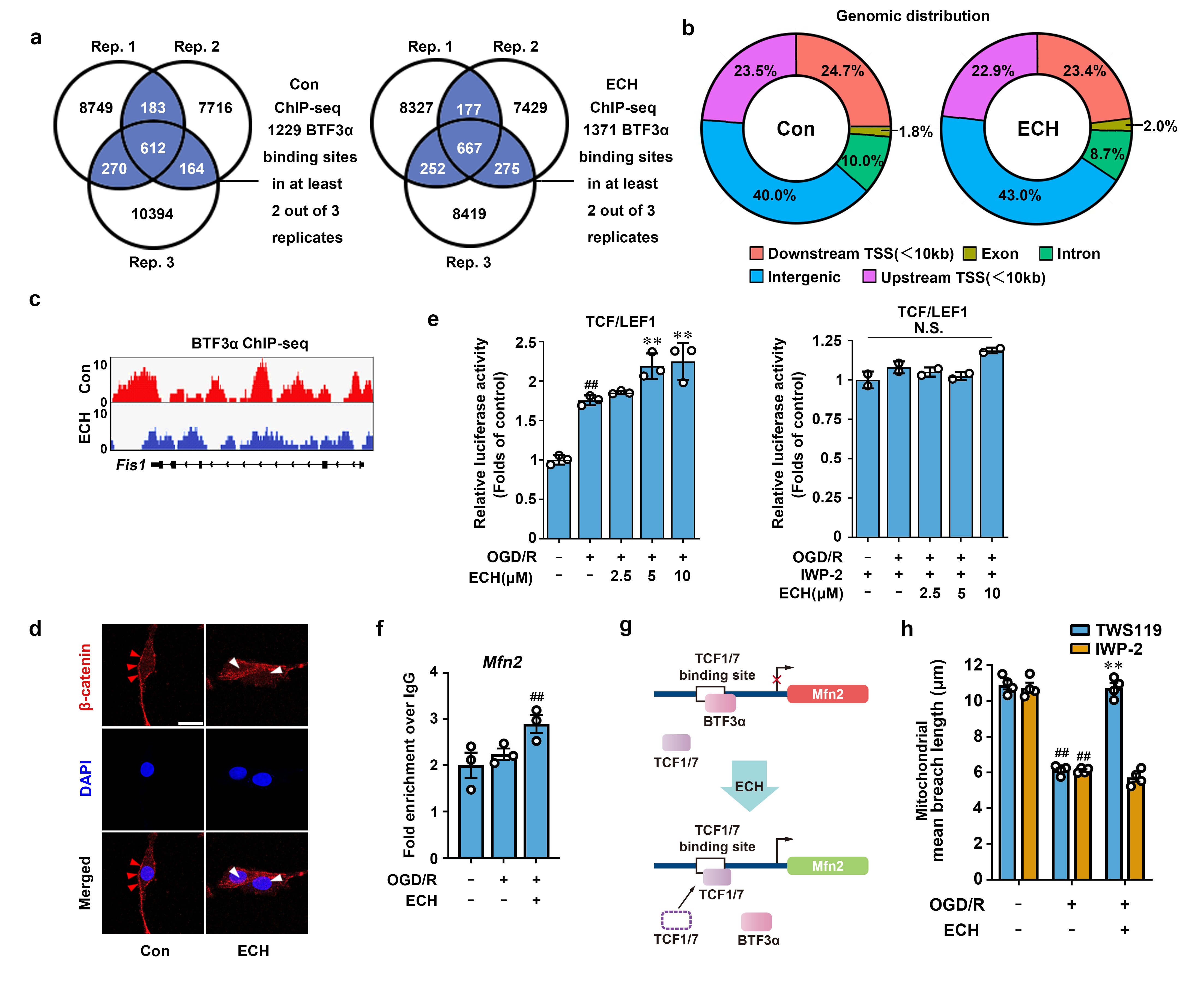
**

**Figure S8. BTF3α facilitates β-catenin-dependent mitochondrial fusion gene *Mfn*2 transcription. a** Venn diagram of BTF3α binding sites in ChIP-seq dataset. **b** Comparison of genomic distribution of BTF3α binding sites with or without ECH treatment. **c** Genome browser view of BTF3α ChIP-seq signal on *Fis1* gene loci. **d** Co-localization of β-catenin (red) and DAPI (blue) was detected by immunofluorescent staining. Arrows (red) indicate cytoplasmic location of β-catenin. Arrows (white) indicate nuclear translocation of β-catenin from the cytoplasm (scale bar: 20 μm). **e** IWP-2 (Wnt inhibitor) significantly blocked ECH-dependent TCF/LEF1 activation. **f** TCF1/7 directly bond to *Mfn2* promoter region upon ECH treatment. **g** Mechanism diagram to clarify the role of TCF1/7 and LEF1 in *Mfn2* transcription. **h** Mitochondrial mean breach length (μm) quantitative analysis of Fig. 5e was performed using ImageJ. Data are expressed as the mean ± SD. ^##^*P* < 0.01 vs control group. ***P* < 0.01 vs OGD/R group. N.S., not significant.

**Figure. S9**

**
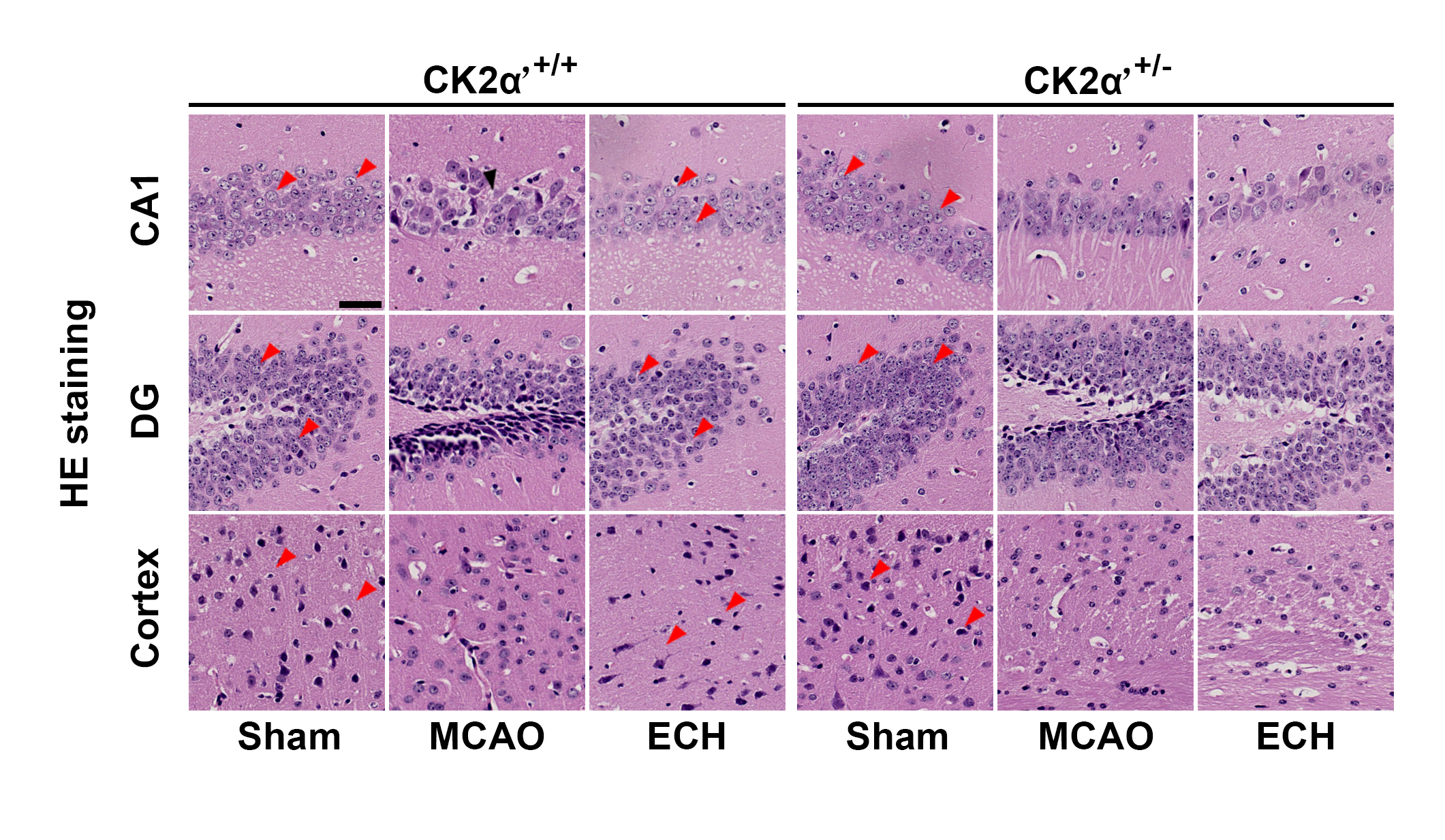
**

**Figure S9. CK2α****’ plays a crucial role on neuronal morphological features.** **a** ECH protected neurons against MCAO insult in CK2α’^+/+^ mice, but showed no effect in CK2α’^+/-^ mice. Neuroprotection in hippocampal and cortical areas were detected by HE staining assays. Arrows indicate normal cells. (scale bar: 50 μm).

**Supplementary tables for methods**

**Supplementary Table 1. siRNA sequences for transfection**

| **siBTF3** | 5’-3’ (sense) GCAGGCACAAGUGCGCAUUTT  5’-3’ (antisense) AAUGCGCACUUGUGCCUGCTT |
| --- | --- |
| **siCK2α’** | 5’-3’ (sense) GAUUACAGCUUGGAUAUGUTT  5’-3’ (antisense) ACAUAUCCAAGCUGUAAUCTT |
| **N.C.** | 5’-3’ (sense) UUCUCCGAACGUGUCACGUTT  5’-3’ (antisense) ACGUGACACGUUCGGAGAATT |

**Supplementary Table 2. Primer pairs for real-time PCR**

| **Gene** | **Sequence** |
| --- | --- |
| ***Mfn2*** | **F**: 5’-GAGTGTCAAGACCGTGAACCA-3’  **R:** 5’-CATCCAGGCAAAACTTATCAATCCA-3’ |
| ***Gapdh*** | **F:** 5’-GGCTCTCTGCTCCTCCC-3’  **R:** 5’-CCGTTCACACCGACCTT-3’ |

**Supplementary Table 3. Primer pairs for ChIP PCR**

| **Gene** | **Sequence** |
| --- | --- |
| ***Mfn2*** | **F**: 5’-TGATCTGGGTAACCATGT-3’  **R:** 5’-CGCCTTTGAGGAAAGAAG-3’ |

**Supplementary Table 4. CK2a’ distinctive binding protein upon ECH treatment**

| **Protein names** | **Gene names** | **Y** | **X** |
| --- | --- | --- | --- |
| Myosin-9 | MYH9 | 1.957133207 | 1.060468523 |
| Protein RCC2 | RCC2 | 1.782680034 | 1.147029784 |
| Protein PRRC2C | PRRC2C | 1.776719559 | 1.211333089 |
| Heterogeneous nuclear ribonucleoprotein A1 | HNRNPA1 | 1.745807622 | 1.208744332 |
| Leucine-rich repeat-containing protein 47 | LRRC47 | 1.706782895 | 1.173467476 |
| Importin subunit alpha-3 | KPNA4 | 1.686153733 | 1.163558045 |
| Translation machinery-associated protein 7 | TMA7 | 1.616424009 | 1.390452412 |
| ATP-dependent RNA helicase DDX18 | DDX18 | 1.599943827 | 1.101604448 |
| 60S ribosomal protein L34 | RPL34 | 1.574505538 | 1.037987759 |
| Ubiquitin carboxyl-terminal hydrolase 10 | USP10 | 1.489615432 | 1.198372342 |
| 60S ribosomal protein L5 | RPL5 | 1.439135238 | 1.274975452 |
| Transcription factor BTF3 | BTF3 | 1.371792141 | 1.60450729 |
| Thioredoxin | TXN | 1.367599681 | 1.229260403 |
| Flap endonuclease 1 | FEN1 | 1.35831239 | 1.25604476 |
| Eukaryotic translation initiation factor 5B | EIF5B | 1.356633329 | 1.310823411 |
| MKI67 FHA domain-interacting nucleolar phosphoprotein | MKI67IP | 1.319387438 | 1.190656021 |
| Cell division cycle 5-like protein | CDC5L | 1.316253692 | 1.205947526 |
| Ubiquitin-conjugating enzyme E2 S | UBE2S | 1.310351417 | 1.06725111 |
